# Rapid Hazard Characterization of Environmental Chemicals Using a Compendium of Human Cell Lines from Different Organs

**DOI:** 10.14573/altex.2002291

**Published:** 2020-06-08

**Authors:** Zunwei Chen, Yizhong Liu, Fred A. Wright, Weihsueh A. Chiu, Ivan Rusyn

**Affiliations:** 1 Interdisciplinary Faculty of Toxicology, College of Veterinary Medicine and Biomedical Sciences, Texas A&M University, College Station, TX, USA;; 2 Department of Veterinary Integrative Biosciences, College of Veterinary Medicine and Biomedical Sciences, Texas A&M University, College Station, TX, USA;; 3 Bioinformatics Research Center, North Carolina State University, Raleigh, NC, USA; 4 Departments of Statistics and Biological Sciences, North Carolina State University, Raleigh, NC, USA

## Abstract

The lack of adequate toxicity data for the vast majority of chemicals in the environment has spurred the development of new approach methodologies (NAMs). This study aimed to develop a practical high-throughput *in vitro* model for rapidly evaluating potential hazards of chemicals using a small number of human cells. Forty-two compounds were tested using human induced pluripotent stem cell (iPSC)-derived cells (hepatocytes, neurons, cardiomyocytes and endothelial cells), and a primary endothelial cell line. Both functional and cytotoxicity endpoints were evaluated using high-content imaging. Concentration-response was used to derive points-of-departure (POD). PODs were integrated with ToxPi and used as surrogate NAM-based PODs for risk characterization. We found chemical class-specific similarity among the chemicals tested; metal salts exhibited the highest overall bioactivity. We also observed cell type-specific patterns among classes of chemicals, indicating the ability of the proposed *in vitro* model to recognize effects on different cell types. Compared to available NAM datasets, such as ToxCast/Tox21 and chemical structure-based descriptors, we found that the data from the five-cell-type model was as good or even better in assigning compounds to chemical classes. Additionally, the PODs from this model performed well as a conservative surrogate for regulatory *in vivo* PODs and were less likely to underestimate *in vivo* potency and potential risk compared to other NAM-based PODs. In summary, we demonstrate the potential of this *in vitro* screening model to inform rapid risk-based decision-making through ranking, clustering, and assessment of both hazard and risks of diverse environmental chemicals.

## 1 Introduction

Most regulatory frameworks for evaluating the safety of drugs and chemicals include a requirement for studies in animals; however, because of the low throughput and high cost of these studies, considerable toxicological information gaps exist for most chemicals in commerce (Locke and Myers, 2011; Taylor et al., 2014; Kavlock et al., 2018). The development of novel non-animal models, both cell-based and computational approaches, to replace animals as the default option in chemical safety evaluation was stimulated by ethical and political pressures (Taylor, 2018), advances in biomedical research and technology, and the need to address the potential hazards from thousands of chemicals in commerce and the environment (NRC, 2007). In the United States and in the European Union, recent changes to the laws that govern the evaluation of commodity and environmental chemicals include provisions that encourage the use of alternative test methods for hazard and risk assessment applications, such as read-across, prioritization, and screening (ECHA, 2016; US EPA, 2018; Taylor et al., 2014). Novel analytical and *in vitro* data, now commonly referred to as new approach methodologies (NAMs), are being used in support of regulatory decisions (Kavlock et al., 2018; Paul Friedman et al., 2020); however, concerns about the limitations of NAMs in decision-making also have been voiced (Gocht et al., 2015; Berggren et al., 2015). The US Environmental Protection Agency (EPA) is developing a strategic plan to reduce the use of vertebrate animals in testing chemical substances and promote the development of alternative test methods; the goal is to eliminate animal testing from regulatory requirements for pesticides and industrial chemicals by 2035 (US EPA, 2019).

The efforts to expand the portfolio of NAMs and test their utility in decision-making are most prominent in the European Union (Daston et al., 2015; Berggren et al., 2015; Desprez et al., 2018; Escher et al., 2019) and the United States (Thomas et al., 2018; Judson et al., 2010a; Kavlock et al., 2018). Data on thousands of chemicals that have been tested in hundreds of lower organism, cell- or molecular-based assays (Kleinstreuer et al., 2014) are publicly available (Williams et al., 2017). These data are used to derive quantitative hazard predictions (Bell et al., 2018; Wetmore, 2015; Pearce et al., 2017; Wambaugh et al., 2015), to address potential data gaps (Chiu et al., 2018; Guyton et al., 2018), and to derive estimates of human health risk when combined with human exposure data or estimates (Sipes et al., 2017; Sirenko et al., 2017; Rotroff et al., 2010; Paul Friedman et al., 2020).

Notwithstanding recent advances in the development of NAMs and publications of a number of case studies on their use for decision-making, many stakeholders, both the industry and the regulators, remain unsure as to what assay(s) should be used to gather data on chemicals or mixtures not currently in ToxCast/Tox21 programs. A traditional approach to development of cell-based models for animal study replacement is to focus on one organ/tissue of concern to the toxicologists, such as the liver (Soldatow et al., 2013), central nervous system (Schmidt et al., 2017), kidney (Su et al., 2016), lung (Lee et al., 2018) or heart (Blanchette et al., 2019). Examples of a successful effort to create targeted sets of *in vitro* assays for a particular decision context are proposals to replace rat uterotrophic (Browne et al., 2015) and Hershberger (Kleinstreuer et al., 2018) assays. In addition, some decision contexts require rapid evaluation of potential chemical hazards in a limited number of assays, such as in response to chemical spills (Judson et al., 2010b; NTP, 2016). Still, little consensus exists with respect to which assays are readily accessible, whether they are reproducible, and how the data shall be analyzed and interpreted.

It also has been reasoned that the pace of transition from animal data to NAMs will depend on the pace at which these new models are optimized to reflect the biology of humans, rather than that of animals (Herrmann et al., 2019). Cancer cell lines, primary cells isolated from non-transplant grade donor tissues, and induced pluripotent stem cell (iPSC)-derived cells are current options for studies of human biology *in vitro*. Of these choices, iPSC-derived organotypic cells are the most physiological and reproducible cell-based model for animal replacement (Anson et al., 2011); however, little toxicological data is available in iPSCs as they are not yet part of ToxCast/Tox21.

In this study, we aimed to conduct an initial test of the performance of a compendium of human *in vitro* models that comprise a small but diverse array of tissues of interest using a representative set of chemicals with known regulatory toxicity values that exemplify major distinct classes of contaminants found on Superfund sites. Specifically, we hypothesized that these cell-based assays can be used for rapid hazard evaluation and thus represent a sensible targeted set of alternative methods for NAM-enabled rapid risk assessment where timely decisions are needed but regulatory toxicity values are lacking. We show that the data from the five-cell-type model was as good or even better in assigning compounds to chemical classes, as compared to either data from large-scale chemical screening programs or chemical structure-based descriptors. In addition, the quantitative data from this model can serve as a conservative surrogate for regulatory decision-making in rapid hazard evaluation scenarios.

## 2 Materials and methods

### Chemicals and biologicals

For our *in vitro* models, we selected four organ/tissue types from which iPSC-derived cells are available from a commercial vendor. iCell hepatocytes 2.0 (Catalogue # C1023), neurons (Catalogue # C1008), cardiomyocytes (Catalogue # CMC-100-010-001) and endothelial cells (Catalogue # C1023), including cell-specific media and supplements, were from Fujifilm Cellular Dynamics (Madison, WI). Pooled human umbilical vein endothelial cells (HUVECs) in EGM-2 medium (Catalogue # CC-2519A) and the EGM^™^-2 BulletKits^™^ (Catalogue # CC-3162) were from Lonza (Walkersville, MD). We selected these cell types because many of the chemicals have been shown to be associated with hepatotoxicity, neurotoxicity, cardiotoxicity, and vascular toxicity. Figure S1^1^ shows the number of published reports for each type of toxicity as identified in a literature review (results are available through the Health Assessment Workspace Collaborative (Shapiro et al., 2018) web portal (see web links in the legend to Fig. S1^1^)). The rationale for cell line selection, metabolic competency of the iCell hepatocyte model, and the justification for selected phenotypes in each cell type are detailed elsewhere (Grimm et al., 2015; Iwata et al., 2017; Sirenko et al., 2014a,b).

Additional reagents used were as follows: CellTiter-Glo^®^ reagent was from Promega (Madison, WI, USA). EarlyTox^™^ Cardiotoxicity Kits (Part# R8211) were from Molecular Devices (San Jose, CA, USA). RPMI 1640 medium, B-27 medium supplement, gentamicin (50 mg/mL), Calcein AM Green, MitoTracker Orange reagent, Hoechst 33342, human fibronectin, and Geltrex^™^ LDEV-Free Reduced Growth Factor Basement Membrane were all from Life Technologies (Grand Island, NY, USA). Recombinant human VEGF was provided by R&D Systems (Minneapolis, MN, USA). Fetal bovine serum (FBS) and Medium 199 were purchased from Fisher Scientific (Waltham, MA, USA). Laminin (Catalogue #L2020-1MG, from Engelbreth-Holm-Swarm murine sarcoma basement membrane) was from Sigma-Aldrich (St. Louis, MO). The authors acknowledge that FBS-free or synthetic FBS-based culture conditions (van der Valk et al., 2018), as well as alternative synthetic basement membrane materials (Nguyen et al., 2017) should be utilized to replace animal-derived products, where appropriate.

The Agency for Toxic Substances and Disease Registry (ATSDR) maintains a priority list of hazardous substances/chemicals^2^ that are frequently detected at the US National Priority List (NPL) sites, also known as “Superfund” sites, and are known human health hazards. From the list of over 300 compounds, we selected 42 chemicals (Tab. 1) based on the review of available information. These compounds represent several classes of pollutants that are ubiquitous in the environment, including polycyclic aromatic hydrocarbons (PAHs, n = 5), inorganic substances (n = 7), phthalates (n = 2), pesticides (n = 20), and other industrial chemicals (n = 8). ATSDR chemical classes are groupings that relate chemicals by similar features based on their structure, uses, physical properties, or other factors. Chemicals were selected for testing based on the following criteria: (i) is listed by ATSDR as priority chemical, (ii) has been evaluated by one or more government agencies and “safe exposure” levels have been established, (iii) was tested in ToxCast/Tox21, and (iv) reverse toxicokinetic and exposure data are publicly available through the EPA dashboard (Williams et al., 2017). Most chemicals were purchased from Sigma-Aldrich), except for heptachlor, heptachlor epoxide, 2,4,5-trichlorophenol, parathion, benzidine and o,p’-DDT, which were from ChemService (West Chester, PA).

### Cell culture and chemical treatments

All cells were cultured in 384-well plates according to the manufacturer’s (Fujifilm Cellular Dynamics or Lonza) recommendations with respect to cell culture media and supplements. Cell density and other cell culture conditions have been previously published for each of these cell types (Grimm et al., 2015; Iwata et al., 2017; Sirenko et al., 2014a,b) and details are included in Text S1^1^. Cells were exposed to test chemicals in descending logarithmic order of concentrations (100, 10, 1, 0.1, and 0.01 μM). Serial dilutions were originally prepared in 100% cell-culture grade DMSO and then further diluted 100-fold in corresponding cell culture medium to yield 4× working solutions in 1% DMSO. The final concentration of DMSO in assay wells following addition of test chemicals was 0.25% (v/v), an amount that was lower than in previous reports where it had no effects on each cell type-derived phenotype (Grimm et al., 2015; Iwata et al., 2017; Sirenko et al., 2014a,b).

### Cytotoxicity assays

Cytotoxicity-related phenotypes in five tested cell types were assessed by high-content live cell imaging after a set exposure time (Tab. 2). Cells were stained with different fluorescent dyes (Hoechst 33342 for nuclei, Calcein AM Green for cytoplasm, and MitoTracker Orange for mitochondria) as detailed in (Grimm et al., 2015; Iwata et al., 2017; Sirenko et al., 2014a,b). Images of all cell culture plates were acquired with ImageXpress Micro Confocal High-Content Imaging System (Molecular Devices) using the DAPI (Hoechst 3342), FITC (Calcein AM Green), and TRITC (MitoTracker Orange) filters at 10× or 20× magnification. Acquired images were processed using the Multi-Wave-length Cell Scoring, Neurite Outgrowth, or Angiogenesis Tube Formation application modules in MetaXpress (Molecular Devices) image processing software, and quantitative data were extracted for concentration-response modeling (see below). In addition, ATP production of iCell neurons and HUVECs was evaluated using CellTiter-Glo assay as described in Text S2^1^.

### Physiologically-relevant phenotype assays

Physiologically-relevant phenotypes of each cell type were evaluated as detailed in Table 2 and reported previously (Grimm et al., 2015; Iwata et al., 2017; Sirenko et al., 2014a,b). Effects on the mitochondrial integrity and intensity of iCell hepatocytes, and neurite outgrowth of iCell neurons were measured using high-content imaging (ImageXpress Micro Confocal High-Content Imaging System, Molecular Devices). Calcium flux reflecting the contract beating of iCell cardiomyocytes was determined by a FLIPR tetra (Molecular Devices) instrument using EarlyTox^™^ Cardiotoxicity Kit as described in Text S3^1^. Effects on angiogenesis of both iCell endothelial cells and HUVECs were measured by 3D cell culture using an extracellular gel matrix and followed by high content imaging as detailed in Text S4^1^.

### Assay quality controls and concentration-response profiling

The qualitative integrity of the screening assays in this study was evaluated using previously established conditions (Grimm et al., 2015). All responses were normalized to the vehicle control (0.25% DMSO-treated wells). Overall, quality control criteria were established to evaluate each cell-based assay based on five parameters (see Tab. S1, S2^1^): (i) variance in replicate wells for two negative controls (vehicle-treated wells and cell medium only), (ii) the difference between two negative controls (vehicle vs cell culture media), (iii) intra- and (iv) inter-plate replicate correlation, and (v) EC_50_ of the positive control chemicals/drugs that were specific for each cell type.

Vehicle control-scaled data for each treatment were fitted to a curve with a nonlinear logistic function to determine point-of-departure (POD) values, defined as the concentrations at which the fitted curve exceeds one standard deviation above or below the mean of vehicle-treated controls, using R software-based script as previously reported (Sirenko et al., 2013). The choice of one standard deviation “benchmark response” was based on the US EPA guidance for dose-response modeling and determination of the point-of-departure values (US EPA, 2012), as well as empirical testing of various thresholds as detailed in (Sirenko et al., 2013), which showed that a choice of one standard deviation generates consistently high classification accuracy.

### Data integration in ToxPi

For data integration and visualization in Toxicological Priority Index Graphical User Interface (ToxPi GUI) (Marvel et al., 2018), we selected 48 phenotypes from all five cell types (Tab. 2). Following the standard ToxPi data protocol, POD values for each phenotype were inversely scaled on a 0–1 scale, with 0 representing the highest POD value in a given data set (i.e., the lowest observed bioactivity) and 1 representing the lowest measured POD value (i.e., the highest observed bioactivity). These scaled POD values were then used as quantitative inputs for bioactivity profiling in ToxPi.

### Clustering and classification analyses

We used two approaches to grouping chemicals based on the biological profiling produced in this study, the bioactivity data from ToxCast/Tox21, and chemical structure-based Morgan fingerprint data. In an unsupervised analysis, chemicals were grouped based on the similarity between the biological/chemical profiling of the chemicals, without prior knowledge of chemical categories. To evaluate the outcome of such grouping, we include a quantitative metric into the unsupervised analysis workflow to assess the correspondence of the outcome to the original categories of each chemical. The details of the unsupervised analysis workflow are described elsewhere (Onel et al., 2019). The Fowlkes-Mallows (FM) index (Fowlkes and Mallows, 1983), a measure of similarity of two clusters, was calculated to enable quantitative comparative assessment between groupings achieved using each dataset to the known chemical categories. The higher the FM index, the more similar the grouping based on *in vitro* or chemical descriptor data was to the “perfect” grouping as shown in Table 1. The FM index ranges from 0.0 (no correspondence) to 1.0 (perfect correspondence). One-sided p-values for the FM index (using the null hypothesis of random assignment) were obtained using a standard z-statistic (Fowlkes and Mallows, 1983) that compares the observed value to the null expectation.

In the supervised analysis, assignments of chemicals to classes (Tab. 1) were used to build classification models, which were then used to predict the class for an unknown chemical. The term “supervised” is a statistical term (Kotsiantis, 2007) referring to models that are trained to perform automatic classification based on the available features, and using the classes as pre-defined groupings. In a supervised analysis, the intent is to identify the features that are best able to distinguish among the classes. For this purpose, the randomForest package in *R* v3.5 was used for class prediction, with 5-fold cross validation implemented in 50 random training/test data splits. The overall prediction accuracy from each database was calculated from cross-validation confusion matrices and the important distinguishing descriptors were further identified. A primary difference between unsupervised and supervised analysis is that the latter focuses on features that best distinguish among existing chemical categorizations.

### Comparison to *in vivo* POD data and margin of exposure estimates

*In vivo* data are still the most commonly used PODs for use in regulatory decision-making, but recent analyses have suggested that NAM-based PODs may be useful as conservative surrogates for *in vivo* values (Paul Friedman et al., 2020). Thus, for the 42 chemicals in this study, we used the *in vivo* PODs from which the regulatory reference doses (RfDs) were derived (POD_RfD_ values) as a benchmark. Specifically, we first compared the POD_RfD_ values to various NAM-based PODs, including the *in vitro* POD derived from this study using iPSC-derived cells and HUVECs, as well as two other *in vitro* data sets: the minimum of the distribution of 50% maximal activity concentration (AC_50_) of high throughput *in vitro* assays in ToxCast database (i.e., most sensitive assay) and conservative POD_NAM_ values reported in (Paul Friedman et al., 2020). In addition, using ExpoCast exposure estimates, we compared margin of exposure (MoE) estimates based on POD_RfD_ values with those based on NAM data. Oral dose-based PODs or exposures were converted to Css (concentration at steady-state)-based values (or *vice versa*) using the high throughput toxicokinetic (httk) (Pearce et al., 2017) R package (v 1.10.1) at the upper 95^th^ percentile for toxicokinetic variability. Due to the limitation of the availability of each data stream, only the chemicals shared in all the databases were taken into consideration for comparison (see details in Tab. S3^1^).

## 3 Results

### 3.1 Screening assays and concentration-response profiling

**Fig. 1:**
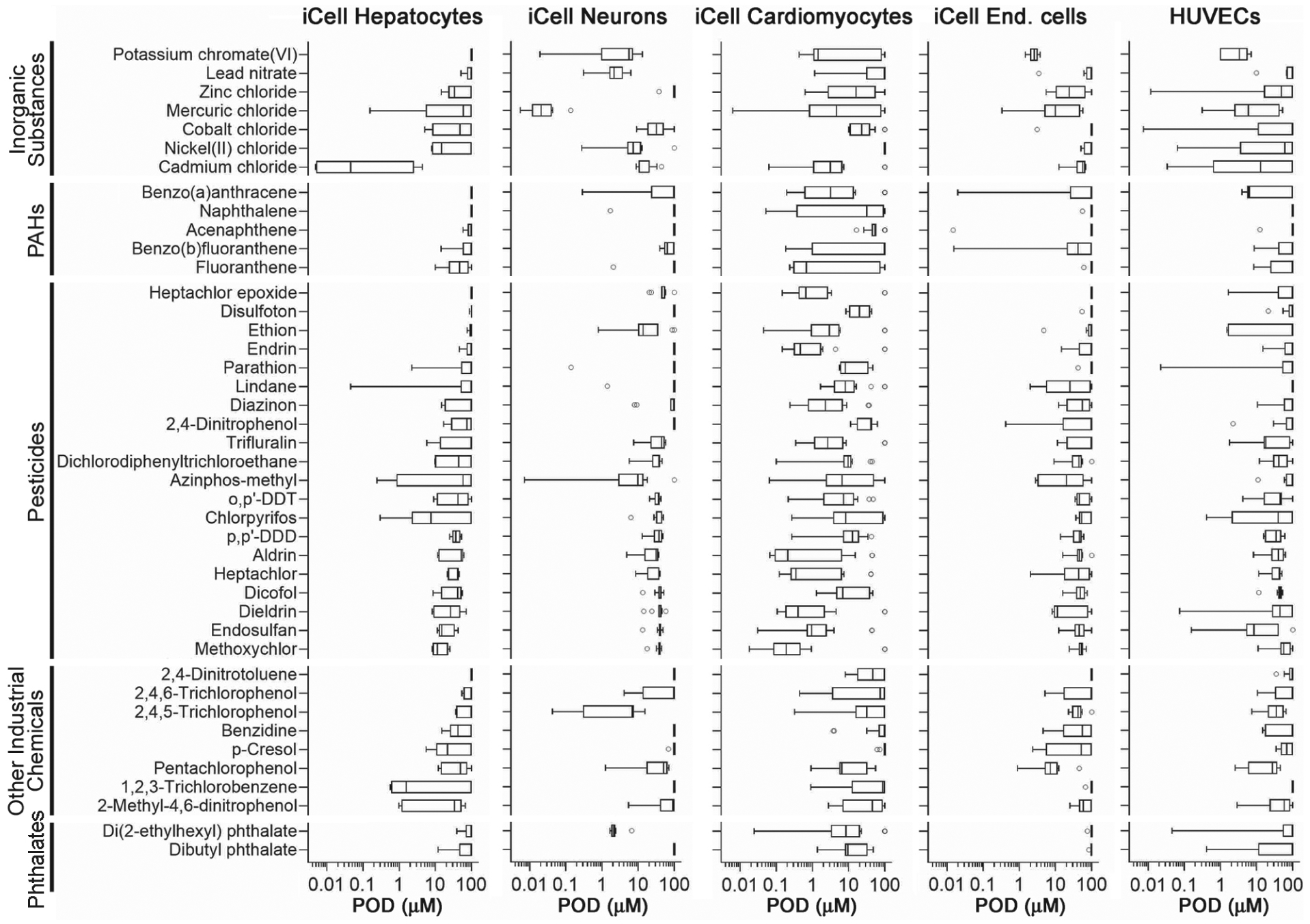
Quantitative analysis of chemical-specific effects in five cell types Box (inter-quartile range and median) and whiskers (min to max) plots show the range of PODs (one standard deviation of vehicle-treated wells) across 48 phenotypes in five cell types (Tab. 2) for each of the 42 Superfund priority list chemicals (Tab. 1). Chemicals were grouped into classes (Tab. 1) and then sorted within a class based on the mean POD values of the phenotypes in iCell hepatocytes.

*In vitro* effects of the test chemicals were evaluated for a wide range of functional and cytotoxicity phenotypes in five human cell types that represent four tissues (Tab. 2). POD values were derived from the concentration-response relationships for a total of 48 phenotypes (see quality control data for each phenotype in Tab. S1 and S2^1^) and plotted (Fig. 1) separately for each cell type. Chemicals are grouped by their chemical class and ranked within each class from least to most bioactive based on the median response in iCell hepatocytes. Both for the individual chemicals and within a chemical class, there was a wide range of potency across all phenotypes. Each chemical had an effect in at least one cell type and no correlation in PODs was evident among cell types (Fig. S2^1^), indicating that the chemicals elicited cell type-specific effects.

**Fig. 2:**
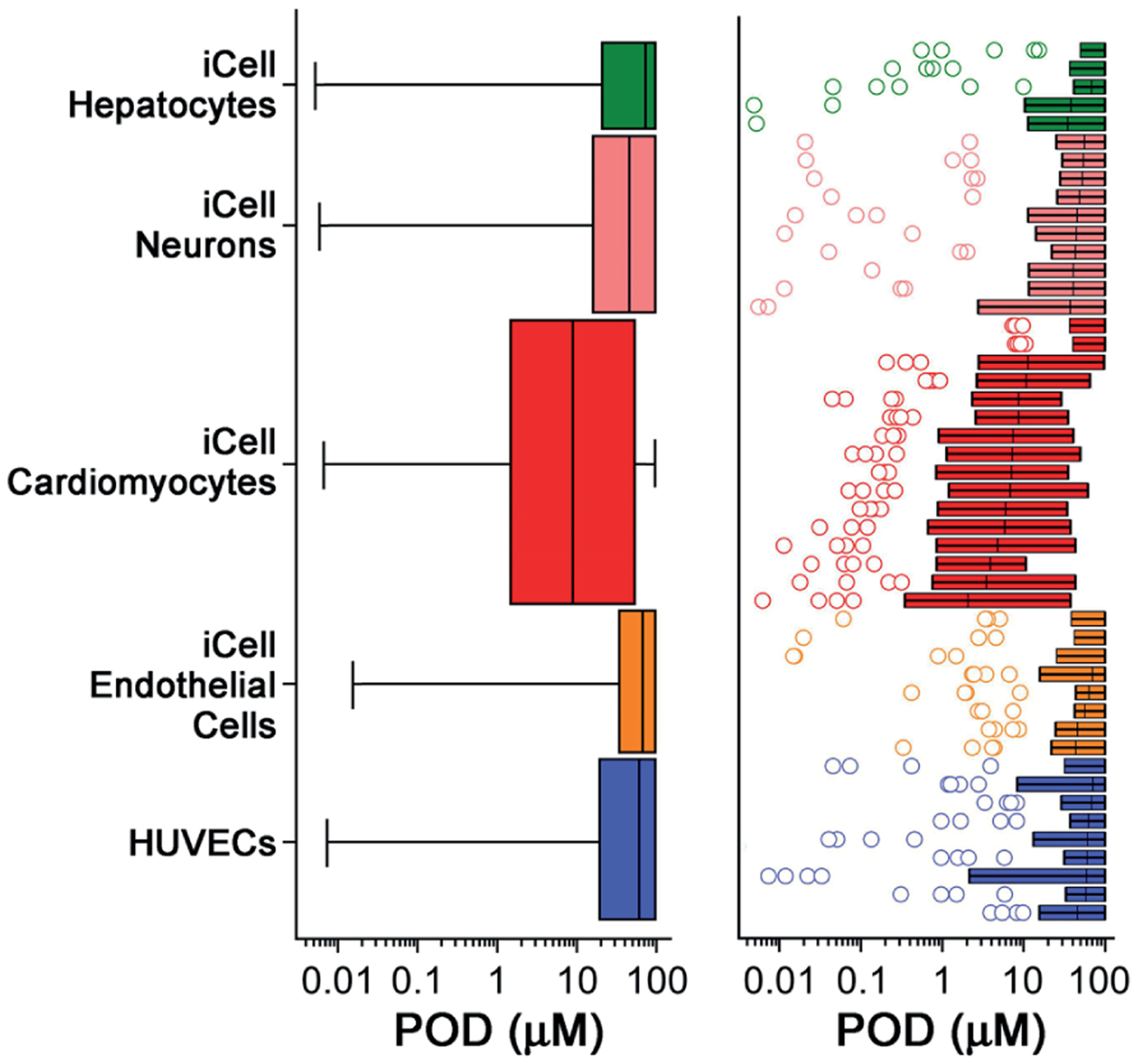
Quantitative analysis of cell-specific effects of the 42 Superfund priority list chemicals The left panel shows box (inter-quartile range and median) and whiskers (min to max) plots of PODs (one standard deviation of vehicle-treated wells) for all 42 tested chemicals (Tab. 1) in each cell type. The size of each box and whiskers plot is proportional to the number of phenotypes evaluated in each cell type (Tab. 2). The right panel shows box (inter-quartile range and median) and whiskers (Tukey) plots of PODs (one standard deviation of vehicle-treated wells) for all 42 tested chemicals (Tab. 1) in each phenotype. Phenotypes are grouped based on the cell type (Tab. 2). Outlier chemicals are shown as circles.

When the PODs were grouped by cell type (Fig. 2), the iCell cardiomyocytes clearly were, on average, the most sensitive to these chemicals. Across the 48 phenotypes included in the analysis, there was a wide range of effects for most of the evaluated chemicals. Not only were there chemicals that had effects at low concentrations, but there was a pronounced shift in the median and inter-quartile range, and for most of the phenotypes that were evaluated (Fig. 2, right panel). In other cell types, few chemicals had pronounced effects while most exhibited effects only at nominal test concentrations above 10 μM. It is noteworthy that fewer effects were observed in metabolically-active iCell hepatocytes (Sirenko et al., 2014b) compared to other cell types. iCell endothelial cells were most resistant to the effects of chemicals tested in this study. In addition, functional effects had significantly lower PODs compared to cytotoxicity phenotypes, indicating higher sensitivity, in all *in vitro* data combined, and in data from iCell hepatocytes, cardiomyocytes and HUVECs (Fig. S3^1^).

### 3.2 Ranking and clustering using ToxPi scores

**Fig. 3:**
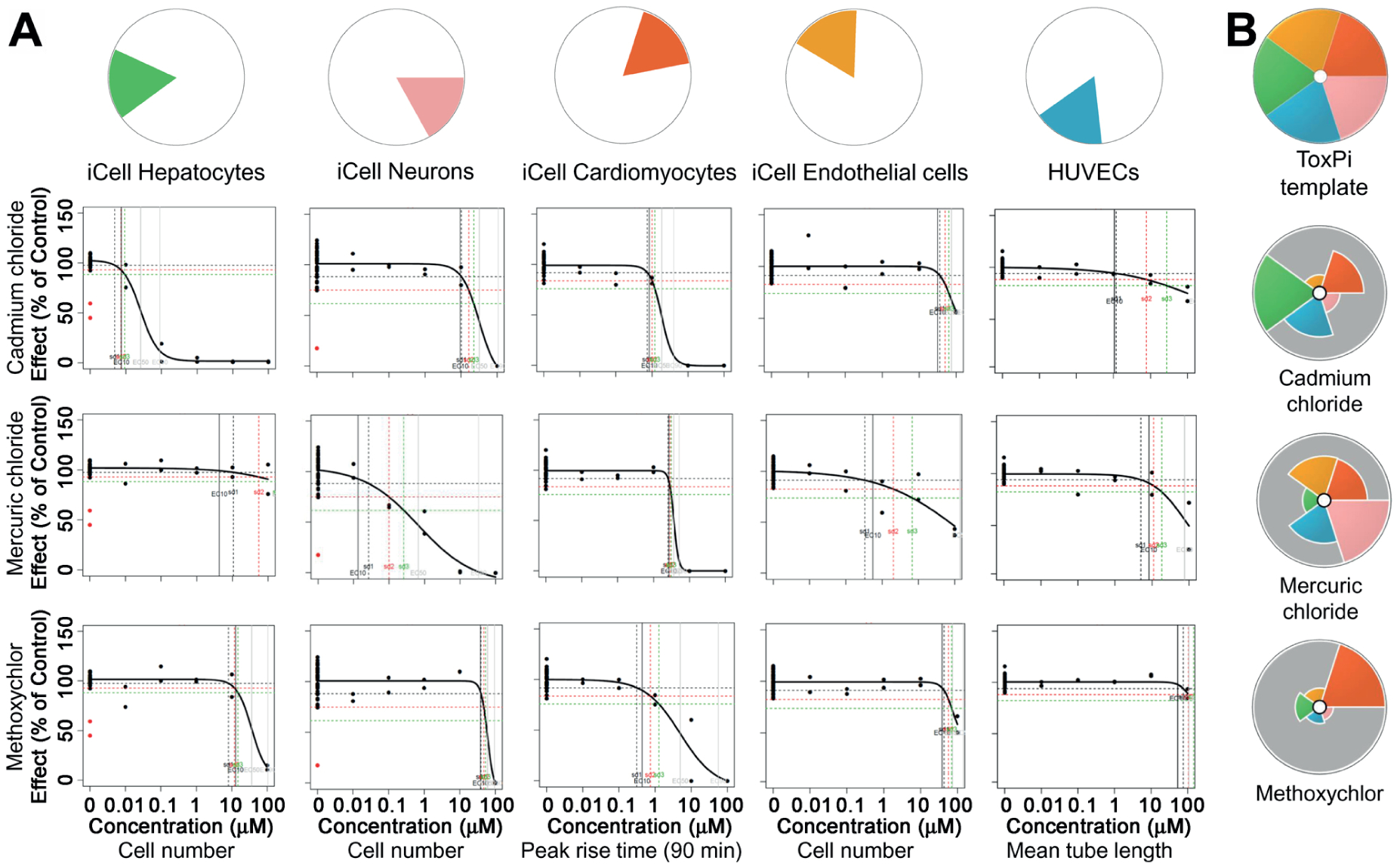
Data integration from concentration-response modeling for each chemical and phenotype using Toxicological Prioritization Index (ToxPi) approach (A) Representative examples of concentration-response fits (lines) to the data (dots) are shown for three chemicals (rows) and five cell-specific phenotypes (columns). Pie chart slices are colored to distinguish effects in each cell type. (B) Examples of ToxPi images for three selected chemicals.

To facilitate interpretation of the data from these experiments that involved five cell types and 48 phenotypes, we aggregated the concentration-response data and PODs derived from *in vitro* screening assays using the Toxicological Priority Index (ToxPi) (Marvel et al., 2018). Each cell type was assigned an individual ToxPi “slice” (Fig. 3A). Specifically, PODs were converted into ToxPi scores as detailed in Section 2 and in Marvel et al. (2018). For each slice, the distance that the arc extends from the origin is proportional to its relative evidence of concern (e.g., longer = greater hazard because of lower POD), and the radial angle (width) indicates its weight in the overall model (in this analysis, data from each cell type were weighed equally). ToxPi scores were further combined into one pie chart to indicate the overall effect of each chemical on all five human cell types. ToxPi for three of the 42 tested chemicals are shown as examples in Figure 3B. Cadmium chloride showed the highest bioactivity (lowest PODs) in iCell hepatocytes compared to the other cell types, resulting in a large green slice in the ToxPi. Mercuric chloride and methoxychlor showed highest effects on iCell neurons and iCell cardiomyocytes, respectively.

**Fig. 4:**
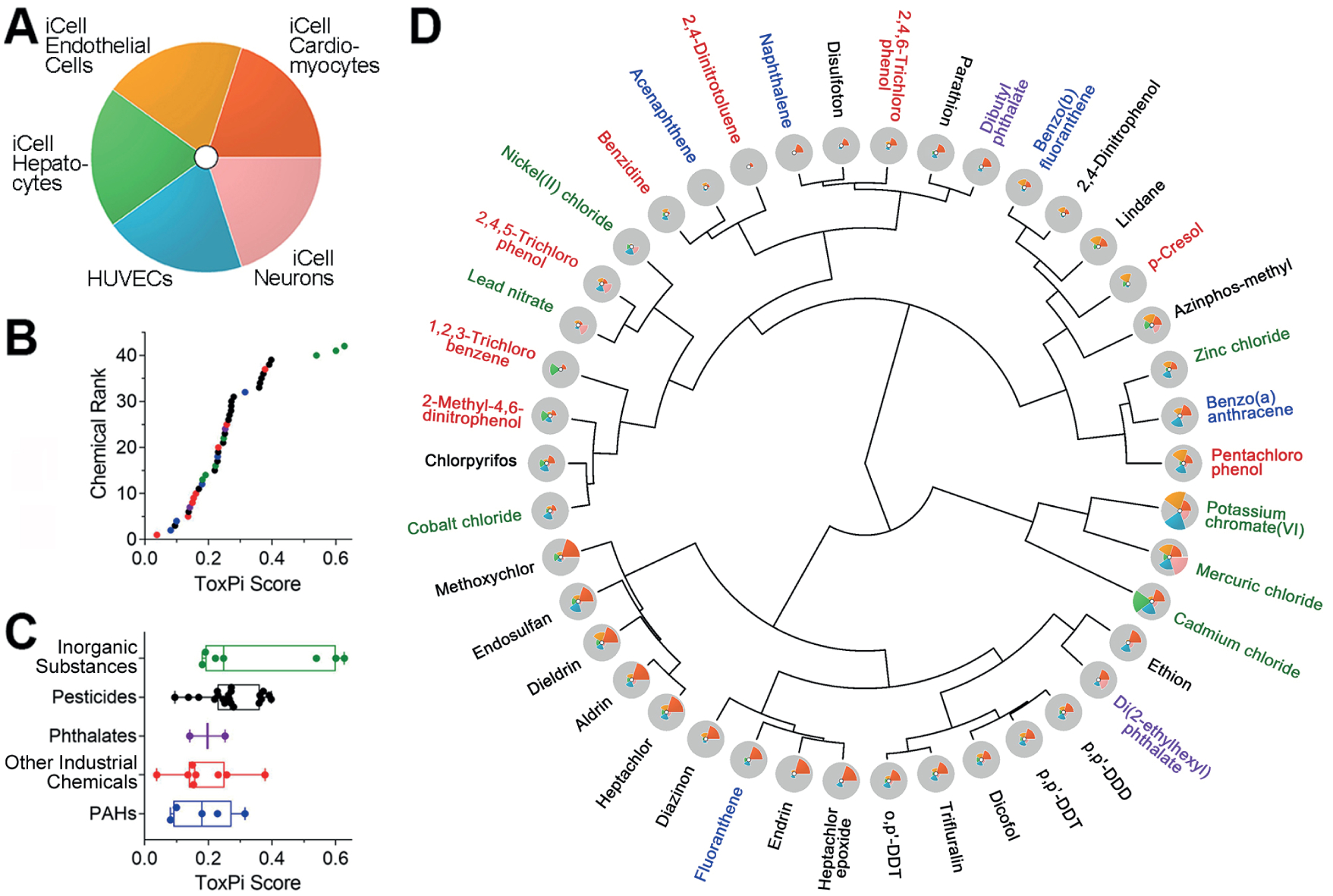
ToxPi analysis-based ranking and clustering of 42 Superfund priority list chemicals based on the effects in five cell types (A) Legend to the ToxPi visualization of the effects on five cell types. (B) Ranking of the tested chemicals based on the overall ToxPi scores. Chemicals are colored based on chemical class. Table S5^1^ contains the data from the ToxPi analysis. (C) Box (inter-quartile range and median) and whiskers (min to max) plots show the range of ToxPi scores for each chemical (dots) for each class. Chemical classes (Tab. 1) were ranked based on the median value. (D) Clustering (Ward’s D method) of 42 Superfund priority list chemicals using ToxPi scores. Chemical names are colored based on chemical class as in panel C.

The overall ToxPi scores for each chemical, reflecting the average of the normalized input scores for each slice of the respective bioactivity profile, were then used as a score to rank and cluster chemicals according to their overall bioactivity (Fig. 4A). ToxPi ranking using quantitative bioactivity data can be used for chemical prioritization (Reif et al., 2010). The 42 tested chemicals were ranked based on the summed effects in the five human cell lines. The three inorganic substances (mercuric chloride, cadmium chloride and potassium chromate) had the highest overall bioactivity score (Fig. 4B). When bioactivity profiles of the individual chemicals were combined into their respective classes, inorganic substances were on average most bioactive, followed by pesticides, phthalates, other industrial chemicals, and PAHs (Fig. 4C, Tab. 3). Furthermore, specific effects of different classes of chemicals on certain cell types were identified. While inorganic substances were bioactive in most cell types, pesticides had the highest bioactivity in iCell cardiomyocytes (Tab. 3, Fig. S4^1^).

Chemicals were also clustered using ToxPi scores and bioactivity profiles (Fig. 4D). This visualization shows that while some compounds are clustered because of their relatively high potency (mercuric chloride, cadmium chloride and potassium chromate), other compounds have similar ToxPi profiles, indicating similarity in their effects on different cell types. For example, DDT-like organochlorine pesticides are clustered closely because of the similarity in both potency and effects across all five cell types. Similarly, other organochlorine pesticides cluster together because they showed the highest relative bioactivity in iCell cardiomyocytes. In addition, phenotype-specific effects of each chemical on each cell type were further identified by clustering chemicals using data on each cell type (Fig. S5^1^). Cadmium chloride exhibited the most pronounced effects on iCell hepatocytes by affecting all phenotypes. Mercuric chloride dominated effects on iCell neurons. Pesticide methoxychlor was the most bioactive in iCell cardiomyocytes. iCell endothelial cells and HUVECs were most affected by potassium chromate.

### 3.3 Bioactivity-based class unsupervised grouping

**Fig. 5:**
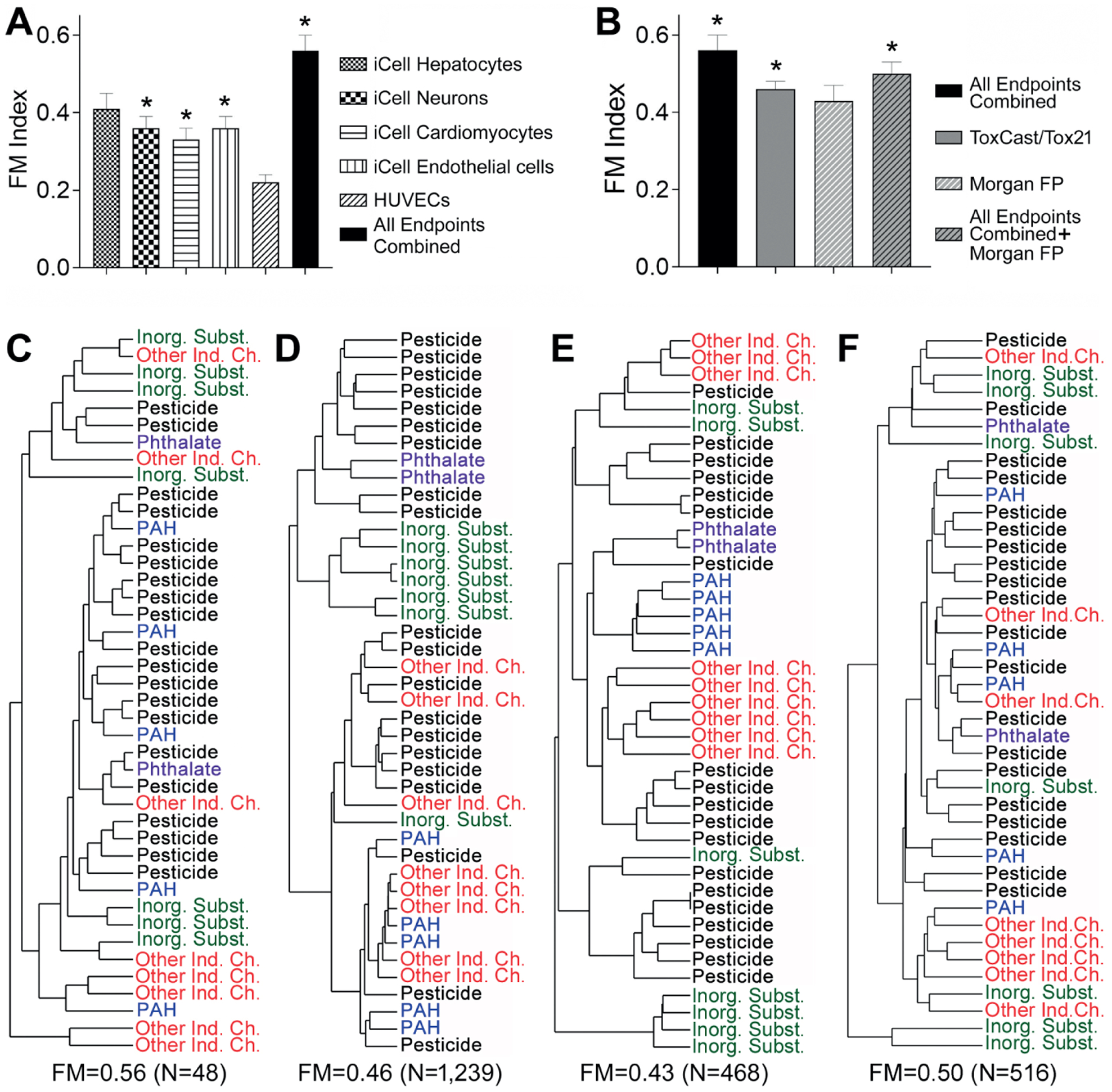
Quantitative analysis of the grouping of 42 Superfund priority list chemicals with various data streams (A) Fowlkes-Mallows (FM) index for clustering of chemicals into five classes (Tab. 1) using *in vitro* data from each cell type, or all data combined. (B) FM index for clustering of chemicals using data in this study (black bar), or other publicly available *in vitro* or chemical descriptors (e.g., Morgan fingerprints [FP]), or a combination thereof. Asterisks (*) indicate that one-sided p-values were < 0.05 for the observed FM index value compared to the null expectation. (C-F) Clustering dendrograms (average Pearson correlation method) for each data stream shown in (B). FM index and the number of variables included in each comparison are shown below each plot. (C) *In vitro* data from this study, all endpoints combined. (D) ToxCast/Tox21 data (as of November 2019). (E) Morgan fingerprints. (F) Morgan fingerprints combined with *in vitro* data from this study. Identity of each chemical in each clustering diagram is listed in Table S6^1^.

Next, we tested how well the bioactivity data on the individual cell type, or in combination, can be used for grouping of tested chemicals into classes. A quantitative comparison of the unsupervised analysis was conducted using the Fowlkes-Mallows (FM) index (Fowlkes and Mallows, 1983; Onel et al., 2019). The results of the clustering were compared to the known chemical groupings (Tab. 1) that were used as a reference. Figure 5A shows that clustering using the bioactivity profiles of the combination of all five cell types resulted in the highest FM index (FM = 0.56) and was highly significant compared to that expected under random permutation (*p* < 0.001). Among the individual cell types, iCell hepatocytes showed the highest FM index (FM = 0.41), albeit it was not significant. Data from HUVECs was least informative in this analysis. Because of the pronounced heterogeneity in the “value” of information from different cell types, we also evaluated whether even smaller sets of cell types may have clustering accuracy approaching the data on all five cell types. We found that a combination of the data from iCell cardiomyocytes and iCell neurons yielded an FM index that was as high as when the data from all five cell types was used (FM = 0.53, Fig. S6^1^).

We also compared the ability of the targeted dataset obtained in this study to group chemicals into classes to that of a larger ToxCast/Tox21 *in vitro* dataset, or chemical structure-based descriptors (Morgan chemical fingerprints). Figure 5B shows that *in vitro* data on 48 phenotypes from five cell types obtained in this study has a higher FM index for grouping of 42 chemicals into five classes compared to other information that is available on these compounds. Figures 5C–E show the individual dendrograms for each of the comparisons in Figure 5B.

### 3.4 Bioactivity-based class supervised grouping

**Fig. 6:**
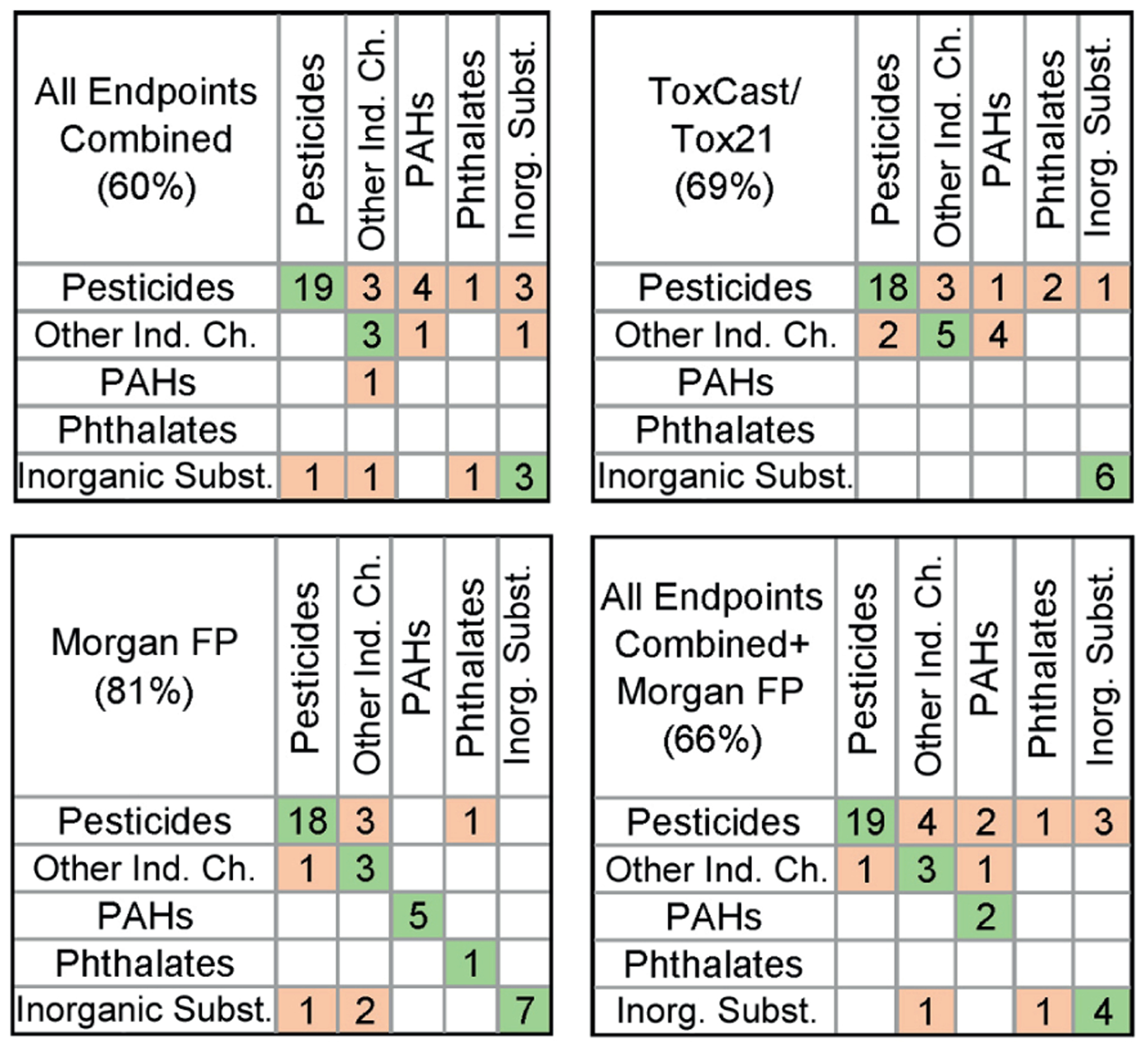
Confusion matrices for chemical classification into five classes using *in vitro* and/or chemical descriptors Known (columns) chemical assignment into each of five classes (Tab. 1) is compared to predicted (rows) class assignment using random forest algorithm with 5-fold cross validation as detailed in Section 2. Classification outcomes for the analyses using data from all phenotypes in this study (top left), ToxCast/Tox21 data (top right), Morgan fingerprints [FP] (bottom left), or data from this study and Morgan FP combined (bottom right) are shown. Accuracy of classification for each dataset is shown in the top left corner of each matrix. Numbers in the cells filled with green (on diagonal) and light pink (off diagonal) indicate the number of chemicals that were classified correctly or misclassified, respectively.

A different type of question that is often asked when using NAM data in decision-making is whether one can use the data obtained in the same set of assays as those for the compounds in a database to classify a new compound into a class. We conducted supervised analyses using a cross-validated random forest algorithm where every test compound was predicted using a classification model. In contrast to the unsupervised analysis, the supervised analysis attempts to train a model to identify the features that are most predictive of existing classification. Figure 6 shows the outcomes of the cross-validated classifications for each data type. Numbers on the top left to bottom right diagonals show correct class prediction, and the numbers off the diagonal show misclassifications and which class the compounds were misclassified into. Overall, the Morgan fingerprints-based classification was superior (81% accurate prediction) when compared to classifications based on either data from this study or ToxCast/Tox21 data (60% and 69%, respectively). It is also noteworthy that the *in vitro* data generated in this study can accurately classify most pesticides into the correct chemical class, whereas ToxCast/Tox21 data classified all inorganic substances correctly. The combination of the *in vitro* data and Morgan fingerprints, or combination of two *in vitro* datasets (Fig. S7A^1^) did not improve prediction accuracy. The accuracy of classification with each type of data was significantly better than random assignment into classes (Fig. S7B^1^). We emphasize that our prediction by supervised analysis was performed using cross-validation, which avoids overfitting inherent in fitting complex prediction models. If the chemical classes used had been truly meaningless, in the sense of “random,” then our reasonably high prediction accuracy values would not have been achieved. The prediction accuracy results suggest that the *a priori* classification is meaningful, and, in contrast to unsupervised analysis, highlight the specific measured biological features that are best able to discriminate among classes, as described below.

**Fig. 7:**
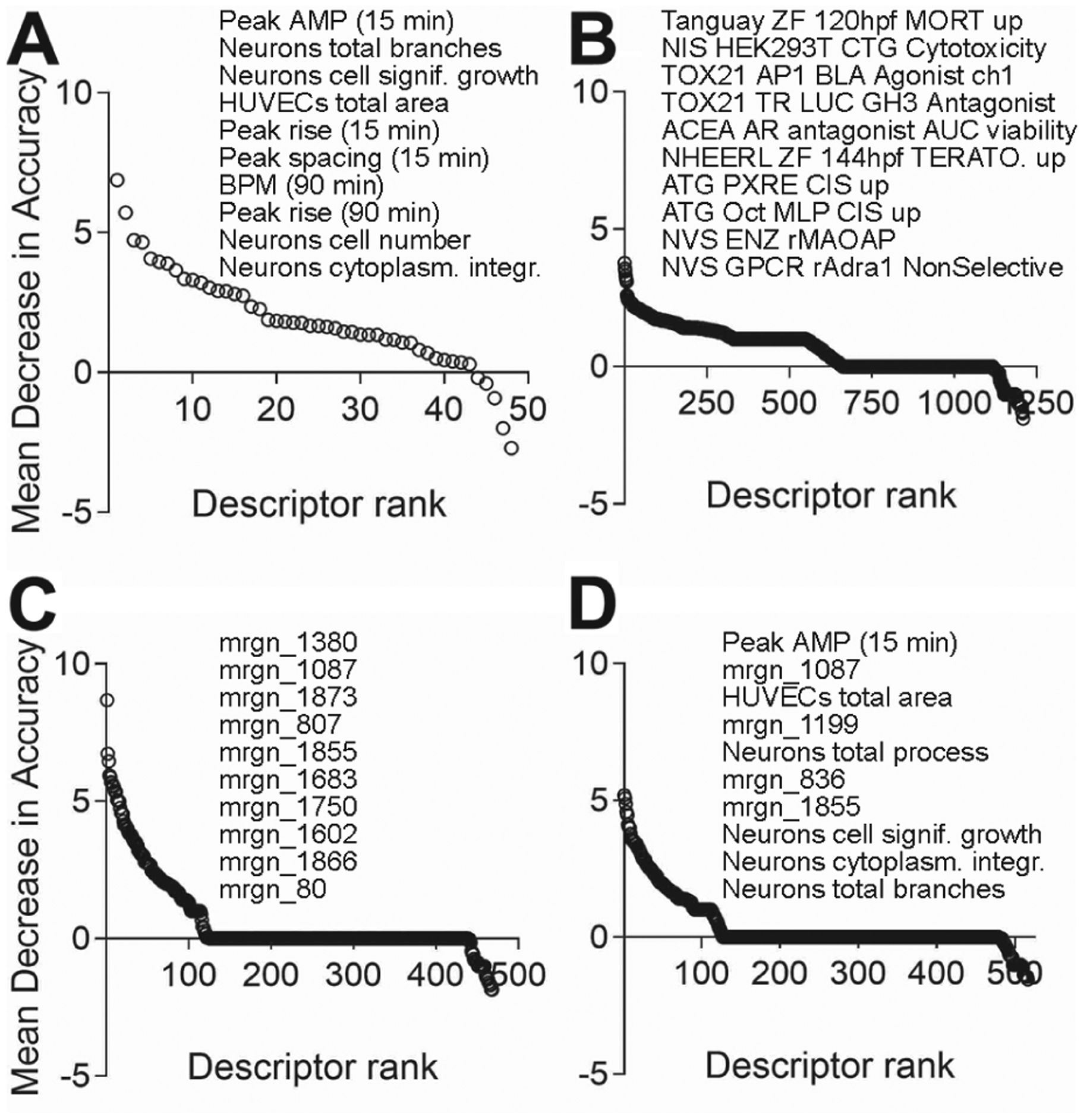
Classification accuracy-contributing phenotypes Importance of the *in vitro* or chemical structure descriptors contributing to the classification accuracy from different data streams (Fig. 6) was analyzed as detailed in Section 2. Top 10 features are listed. (A) *In vitro* data from this study. (B) ToxCast/Tox21 data. (C) Morgan fingerprints. (D) Morgan fingerprint combined with *in vitro* data from this study.

The supervised classification analysis, where every test compound was predicted using a classification model, can also be examined for information on the “most informative” features (i.e., features that are most predictive of existing classification) on which the models were developed. The top 10 most informative features from each dataset, i.e., phenotypes that contributed the most to the accuracy of the classification, are shown in Figure 7. Interestingly, for the *in vitro* data generated in this study, 5 of the top 10 most informative descriptors were functional phenotypes from iCell cardiomyocytes, followed by phenotypes from iCell neurons (Fig. 7A). For ToxCast/Tox21 data, the descriptors in the top 10 included largely disparate data from a wide range of models, i.e., from zebrafish, to cytotoxicity, to reporter assays (Fig. 7B). While Morgan fingerprints are difficult to interpret directly (Fig. 7C), a combination of bioactivity and chemical structure data showed that chemical descriptors do not dominate the list of informative features, and that *in vitro* data may be equally informative (Fig. 7D).

### 3.5 Comparison to *in vivo* POD data and margin of exposure estimates

**Fig. 8:**
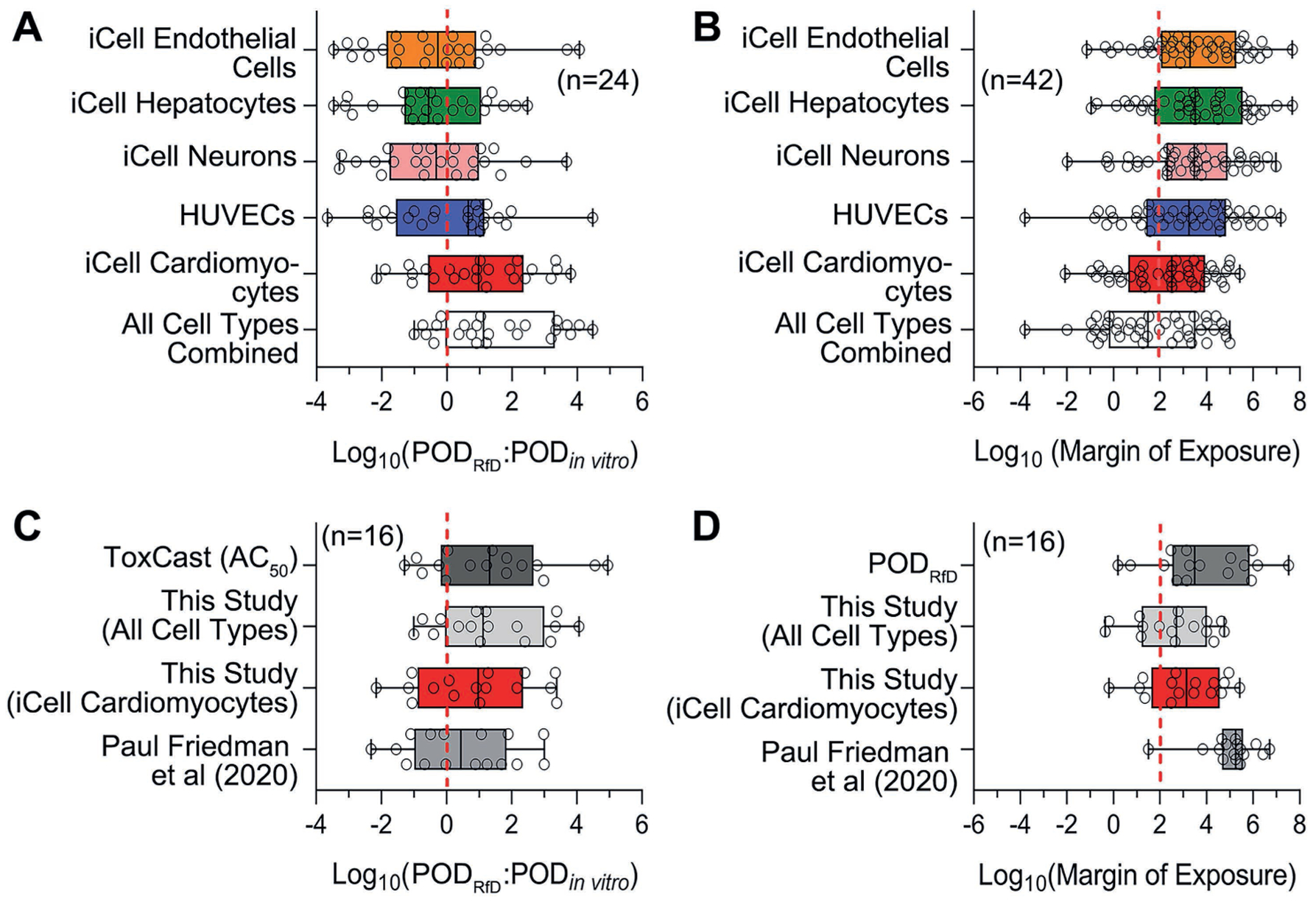
POD data comparison across different *in vitro* and *in vivo* datasets and margin of exposure estimates Minimum of *in vitro* PODs generated from each cell type and all cell types combined in this study were compared to *in vivo* POD derived from Reference dose (A). Margins of exposure were calculated based on *in vitro* PODs from this study and the estimated exposure levels (B). The ratio between *in vivo* and *in vitro* (C), and the margins of exposure (D) were further compared across different datasets. All of the ratio outputs were log transformed for comparison; *n* represents the number of chemicals from 42 Superfund priority list chemicals covered by different datasets for comparison and detailed in Table S3^1^.

It has recently been proposed that NAM-based PODs can serve as conservative surrogates for traditional *in vivo* PODs (Paul Friedman et al., 2020). Thus, we first compared various NAM-based PODs, including those based on our five cell types, to the regulatory PODs used as the basis for RfD toxicity values (POD_RfD_). For our *in vitro*-based PODs, we used either the most sensitive POD for each cell type or the most sensitive POD across all cell types combined (Fig. 8). As shown in Figure 8A, only when all cell types are combined do our *in vitro* PODs represent a conservative surrogate for the POD_RfD_, with only 25% of our *in vitro* PODs being higher than the corresponding POD_RfD_, and those remaining 25% being within 10-fold of the *in vivo* value. In contrast, as shown in Figure 8C, only the approach of using the minimum (most sensitive) ToxCast AC_50_ has similarly conservative results, whereas cardiomyocytes alone and the POD_NAM_ from (Paul Friedman et al., 2020), which is a lower 5^th^ percentile, had a substantial number of “unconservative” results. Note that these results appear to contrast with those reported by (Paul Friedman et al., 2020) because they used *in vivo* PODs from ToxRefDB, whereas we used the *in vivo* PODs that supported regulatory RfD toxicity values (Wignall et al., 2014).

A related comparison was with respect to the resulting screening-level risk characterization using a Margin of Exposure (MoE) approach. Specifically, we used a MoE benchmark of < 100 as an indication of “potential concern.” As shown in Figure 8B, more than half of the chemicals have implied MoEs less than a benchmark of 100 when using all cell types combined, with similar results for cardiomyocytes, but far fewer chemicals are suggested to be of “potential concern” when using other cell types. In Figure 8D, when restricting to chemicals common across different NAM-based approaches, we find that the POD_RfD_-based “ground truth” suggests that only 2/16 chemicals are of “potential concern.” Using only iCell cardiomyocytes, or using all cell types, results in a more conservative estimate of 4 to 5/16 chemicals, with the median MoE being slightly more conservative than the *in vivo*-based MoE. In contrast, using the POD_NAM_ from (Paul Friedman et al., 2020) results in an “unconservative” estimate of only 1/16 chemicals of potential concern, with the median MoE being much higher (implying “safer”) than the *in vivo*-based MoE.

Overall, for this limited dataset, our PODs derived from high throughput *in vitro* data from five human cell types performed well as a conservative surrogate for regulatory *in vivo* PODs and were less likely to underestimate *in vivo* potency and potential risk compared to other NAM-based PODs.

## 4 Discussion

It is widely recognized that the future of regulatory toxicology lies in high-throughput *in vitro* assays and computational models based on human biology, rather than in continued testing in laboratory animals (NRC, 2007; National Academies of Sciences Engineering and Medicine, 2017). A wide array of both biological and computational tools is available to probe human function and disease at the molecular level through the transcriptome, epigenome, proteome and metabolome (Nielsen, 2017). Many thousands of immortalized cell lines collected from various tissues and individuals are now used in toxicological research (Chiu and Rusyn, 2018). There are large databases of publicly available biological data that can be explored to develop hypotheses about how chemicals, genes, and diseases may be connected (Miller, 2016; Davis et al., 2019; Williams et al., 2017). There are genetically diverse mammalian and non-mammalian models, *in vivo* and *in vitro*, that are used for toxicological research (Zeise et al., 2013). Complex human biology is being replicated in multicellular perfused microphysiological systems that mimic certain tissue functions (Marx et al., 2020). It appears that the field of regulatory science has finally overcome the long-lamented challenge of shortage of information for decisions on chemical safety (Lutter et al., 2013).

Alas, the quantity of the information now available is yet to be translated into actual examples of using these data in various decision contexts beyond now well-accepted screening-level, risk-based chemical prioritization (Harrill et al., 2019; Paul Friedman et al., 2020), or filling data gaps (Guyton et al., 2018). For new chemicals, complex substances, or mixtures, what is a sensible compendium of *in vitro* and *in silico* models that may satisfy the data requirements for a particular decision context? A number of examples have been published recently to address this question, especially in the context of grouping and read-across (De Abrew et al., 2019; Zhu et al., 2016; Escher et al., 2019). Indeed, it is critically important to establish both the strengths and limitations of cell-based *in vitro* screening methods, so that promising NAMs can be generated and used for decision-making in human and environmental health.

This study, even though primarily focused on an *in vitro* model that can be used for rapid hazard assessment, adds to the overall body of recent evidence on the topic of the utility of NAMs. We aimed to test performance of a small set of human *in vitro* models that represent a diverse array of tissues of interest to regulatory toxicologists. We took advantage of recently developed reproducible and physiologically-relevant human *in vitro* models derived from iPSCs (Li and Xia, 2019; Anson et al., 2011), models that are excellent replacements for animal tests and for which detailed methods and metrics of reproducibility have been established (Sirenko et al., 2013, 2014a,b; Grimm et al., 2018; Iwata et al., 2017; Klaren and Rusyn, 2018). We posited that commercially-available iPSC-derived cells are poised for wider use, replacement of animal studies, and inter-comparison of the outcomes in a rigorous and reproducible manner (Anson et al., 2011). Presence of advanced cellular functions and absence of genetic drift because of repeated passaging, both problems of cancer cell lines, are advantages of iPSC-derived differentiated cells in toxicity testing (Kim et al., 2019). Our hypothesis was that these cell-based models, when probed for both physiological and toxicological effects of chemicals, can be used for rapid hazard evaluation and thus represent a sensible targeted set of alternative methods for NAM-enabled decisions, especially under conditions of rapid evaluations such as emergency response (Judson et al., 2010b).

Even though this study is not the first to attempt to probe the ability of a small dataset to group and classify diverse environmental chemicals, a number of important learnings have emerged. First, our comparison of cells representing various tissue types showed that iPSC-derived cardiomyocytes may be among the cell types that are most sensitive to effects across various chemical classes. This is noteworthy because iCell cardiomyocytes can be used as a highly reproducible *in vitro* model that faithfully replicates many *in vivo* cardiotoxic phenotypes (Grimm et al., 2018). Our previous studies showed that environmental chemicals have adverse effects on cardiomyocytes, similar to many known cardiotoxic drugs (Sirenko et al., 2017; Burnett et al., 2019; Blanchette et al., 2019); however, it is noteworthy that this metabolism-limited cell type was most affected by the diverse set of Superfund priority chemicals from different classes.

Second, the fact that the chemicals tested in this study showed very divergent effects across multiple cell types, leading to distinct class-specific bioactivity profiles that can be used to group substances, also strongly supports the need for tissue diversity of *in vitro* models. Moreover, when used for NAM-based risk characterization, multiple cell types together performed better than any individual cell type for ensuring that the risk is not underestimated. These findings suggest that when testing is not meant to be mechanism- or effect-based, inclusion of cells from multiple tissues should be a design principle for *in vitro* test batteries that are to be used as NAMs. Such tissue-diverse data should also increase confidence in the “biological coverage” of *in vitro* NAMs.

Third, we observed that *in vitro* bioactivity data may be as good as or, in some cases better than, chemical descriptors for grouping of chemical substances into classes. In addition, important synergies are realized when biological and chemical descriptors are combined. These findings are in line with previous observations that chemical-biological data are most powerful for grouping (Low et al., 2011, 2013, 2014), as well as that they are most interpretable by the decision-makers (Zhu et al., 2016).

Finally, we found that a limited set of *in vitro* data may be equally or even more informative that the much larger datasets from large-scale chemical screening programs (Thomas et al., 2018). Overabundance of NAM data is not necessarily a recipe for more accurate prediction, as has been shown for various types of biological (Kreutz et al., 2013) and chemical (Fourches et al., 2015) data. One approach to dealing with such “big data” problems is to apply variable selection (Knudsen et al., 2013) or deep learning (Grapov et al., 2018) algorithms to uncover meaningful “signals” in large datasets. Regretfully, these exercises seldom have resulted in selection of a reasonably small set of assays/endpoints that are reasonably accurate for prediction and do not require extensive and lengthy experimentation. Only recently, influential examples have emerged of how a small set of assays can be used to replace a specific animal test (Kleinstreuer et al., 2018; Browne et al., 2015). On the other hand, the data from our study performed at least as well, if not better, than larger NAM datasets, not only for grouping of chemicals into classes, but also in serving as surrogate NAM-based PODs for rapid risk characterization. Additional confidence in these results could be obtained by evaluating a larger set of ToxCast/Tox21 chemicals.

Notwithstanding the need for diverse high-throughput *in vitro* data streams to rapidly inform hazard identification and to fill the knowledge gap for chemicals with minimum toxicity data, challenges remain about their use in prioritization and screening level assessment strategies as well as tradeoffs between speed and uncertainty (Paul Friedman et al., 2020). For instance, while high throughput screening data could play key roles in decision-making for emergency response, there are many limitations with respect to predicting chemical fate and effects in the environment, challenges that might lead to potentially missed hazards (Ginsberg et al., 2019). Furthermore, there is also uncertainty in the extrapolation from *in vitro* bioactivity to *in vivo* toxicity (Bell et al., 2018), and gaps exist in the cell-based *in vitro* screening and potential effects on human health since most cell assay endpoints are still related to cytotoxicity and non-specific effects (Judson et al., 2016). Overall, however, our findings support the notion that the field of *in vitro* toxicology and NAM implementation would be well served by agreeing on a reasonably small subset of differentiated, human cell-based models with both cytotoxicity-based and functional readouts that can be used in different decision contexts.

## Supplementary Material

Supplemental Information

## Figures and Tables

**Tab. 1:** Superfund priority chemicals used in this study

ATSDR Chemical class	Chemical name	CAS number	Chemical formula	ATSDR rank (2017)
**Inorganic substances**	Lead nitrate	10099-74-8	PbCl_2_	2
	Mercuric chloride	7487-94-7	HgCl_2_	3
	Cadmium chloride	10108-64-2	CdCl_2_	7
	Potassium chromate (VI)	7789-00-6	K_2_CrO_4_	17
	Cobalt chloride	7646-79-9	CoCl_2_	51
	Nickel chloride	7718-54-9	NiCl_2_	57
	Zinc chloride	7646-85-7	ZnCl_2_	75
**Polycyclic aromatic hydrocarbons (PAHs)**	Benzo(b)fluoranthene	205-99-2	C_20_H_12_	10
	Benzo(a)anthracene	56-55-3	C_18_H_12_	38
	Naphthalene	91-20-3	C_10_H_8_	81
	Fluoranthene	206-44-0	C_16_H_10_	138
	Acenaphthene	83-32-9	C_12_H_10_	171
**Pesticides**	*p,p’*-DDT	50-29-3	C_14_H_9_Cl_5_	13
	Dieldrin	60-57-1	C_12_H_8_Cl_6_O	18
	Aldrin	309-00-2	C_12_H_8_Cl_6_	25
	*p,p’*-DDD	72-54-8	C_14_H_10_Cl_4_	26
	Heptachlor	76-44-8	C_10_H_5_Cl_7_	28
	Lindane	58-89-9	C_6_H_6_Cl_6_	34
	Disulfoton	298-04-4	C_8_H_19_O_2_PS_3_	37
	Endrin	72-20-8	C_12_H_8_Cl_6_O	40
	Diazinon	333-41-5	C_12_H_21_N_2_O_3_PS	41
	Endosulfan	115-29-7	C_9_H_6_Cl_6_O_3_S	44
	Heptachlor epoxide	1024-57-3	C_10_H_5_Cl_7_O	47
	*o,p’*-DDT	789-02-6	C_14_H_9_Cl_5_	53
	Methoxychlor	72-43-5	C_16_H_15_Cl_3_O_2_	55
	Chlorpyrifos	2921-88-2	C_9_H_11_Cl_3_NO_3_PS	64
	2,4-dinitrophenol	51-28-5	C_6_H_4_N_2_O_5_	89
	Ethion	563-12-2	C_9_H_22_O_4_P_2_S_4_	99
	Azinphos-methyl	86-50-0	C_10_H_12_N_3_O_3_PS_2_	131
	Dicofol	115-32-2	C_14_H_9_Cl_5_O	145
	Parathion	56-38-2	C_10_H_14_NO_5_PS	148
	Trifluralin	1582-09-8	C_13_H_16_F_3_N_3_O_4_	157
**Other industrial chemicals**	Benzidine	92-87-5	C_12_H_12_N_2_	30
	Pentachlorophenol	87-86-5	C_6_Cl_5_OH	54
	2,4,6-trichlorophenol	88-06-2	C_6_H_2_Cl_3_OH	85
	2,4-dinitrotoluene	121-14-2	C_7_H_6_N_2_O_4_	98
	2-Methyl-4,6-dinitrophenol	534-52-1	C_7_H_6_N_2_O_5_	100
	1,2,3-Trichlorobenzene	87-61-6	C_6_H_3_Cl_3_	137
	2,4,5-Trichlorophenol	95-95-4	C_6_H_2_Cl_3_OH	142
	*p*-Cresol	106-44-5	C_7_H_8_O	175
**Phthalates**	Dibutyl phthalate	84-74-2	C_16_H_22_O_4_	58
	Di(2-ethylhexyl) phthalate	117-81-7	C_24_H_38_O_4_	77

**Tab. 2:** *In vitro* toxicity phenotypes evaluated in this study

Cell type^a^	iCell hepatocytes	iCell neurons	iCell cardiomyocytes^b^	iCell endothelial cells^c^	HUVEC^c^
**Catalog #**	C1023	C1008	CMC-100-010-001	C1114	CC-2519A
**Time point**	24 h	72 h	15 or 90 min	18 or 24 h	18 or 24 h
**Functional phenotypes**	– Mitochondrial integrity – Mitochondrial intensity	– Total outgrowth – Mean outgrowth – Total process – Total branches – Cells with significant growth	– Beats per minute – Peak amplitude – Peak spacing – Peak width – Peak rise time – Peak decay time – Decay to rise ratio	– Total tube length – Mean tube length – Total tube area	– Total tube length – Mean tube length – Total tube area
**Cytotoxicity phenotypes**	– Cell number – Nuclei intensity – All cell mean area	– Cell number – Mitochondrial integrity – Cytoplasmic integrity – Total cells body area – ATP^d^	– Cell number – Mitochondrial integrity	– Cell number – Mitochondrial integrity – Mitochondrial intensity – Cytoplasmic integrity – Nuclei mean area	– Cell number – Mitochondrial integrity – Mitochondrial intensity – Cytoplasmic integrity – Nuclei mean area – ATP^d^

See Table S4^1^ for detailed description of each phenotype.

a iCell lines were purchased from FujiFilm Cellular Dynamics, HUVEC cell line was purchased from Lonza.

b Cytotoxicity phenotypes were measured in iCell cardiomyocytes at 90 min.

c Cytotoxicity phenotypes were measured in iCell endothelial cells and HUVEC at 24 h.

d CellTiter-Glo^®^ assay.

**Tab. 3:** Ranges in ToxPi scores for each chemical class and cell type

Cell type	PAHs	Pesticides	Inorganic substances	Other industrial chemicals	Phthalates
**iCell hcpatocytos**	0–0.14	0–0.32	0–0.88	0–0.45	0.026–0.03
**iCell neurons**	0–0.11	0–0.37	0.01–1	0–0.47	0–0.46
**iCell cardiomyocytes**	0.10–0.55	0.18–0.78	0–0.50	0.01–0.34	0.37–0.42
**iCell endothelial cells**	0.02–0.27	0–0.38	0.04–0.72	0–0.49	0.005–0.009
**HUVECs**	0–0.41	0–0.38	0.10–0.75	0–0.36	0.13–0.18
**Overall (combination of all phenotypes)**	0.08–0.32	0.10–0.39	0.18–0.63	0.04–0.38	0.14–0.25

## Footnotes

1 doi:10.14573/altex.2002291s

2 http://www.atsdr.cdc.gov/spl
