# Supplementary material for: Rapid Hazard Characterization of Environmental Chemicals Using a Compendium of Human Cell Lines from Different Organs
Source: ALTEX. Author manuscript; Available in PMC 2021 Dec 8. (PMC7941183; doi:10.14573/altex.2002291)
Supplement: Supplemental Information [file NIHMS1670765-supplement-Supplemental_Information.docx]

**Supporting information:**

**Rapid hazard characterization of environmental chemicals**

**using a compendium of human cell lines from different organs**

Zunwei Chen,^1,2^ Yizhong Liu,^1,2^ Fred A. Wright,^3,4^ Weihsueh A. Chiu, ^1,2^ Ivan Rusyn^1,2,^*

^1^Interdisciplinary Faculty of Toxicology and ^2^Department of Veterinary Integrative Biosciences, College of Veterinary Medicine and Biomedical Sciences, Texas A&M University, College Station, TX 77843

^3^Bioinformatics Research Center and ^4^Departments of Statistics and Biological Sciences, North Carolina State University, Raleigh, NC 27695

*To whom correspondence should be addressed:

Ivan Rusyn, MD, PhD, Department of Veterinary Integrative Biosciences, Texas A&M University, College Station, TX 77843; irusyn@cvm.tamu.edu; (979) 458-9866

**Text S1: Detailed cell culture procedures**

*iCell hepatocytes 2.0* (Grimm et al. 2016): vials of hepatocytes were thawed for 3 min at 37℃ in a water bath and subsequently resuspended in RPMI medium containing 2% (v/v) iCell hepatocyte medium supplement, 0.1 μM dexamethasone, 2% (v/v) B27 supplement, 25 μg/ml Gentamicin, and 20 ng/ml Oncostatin-M. Following microscopic evaluation of the cell density, the suspension was further diluted to a final concentration of 6.72 × 10^5^ cells/ml. 25 μl of this suspension was then added to each well on collagen I coated 384-well plates (Corning, Product# 354664), yielding a final cell density of 16,800 cells per well. Plates were initially kept at room temperature for 30 min and then transferred to an incubator set at 37°C and 5% CO2. After four hours of incubation, the plating medium was replaced with 25 μl fresh medium, a step that was repeated daily for four days. On day five, the plating medium was exchanged with 25 μl per well maintenance medium, consisting of RPMI containing 2% (v/v) iCell hepatocyte medium supplement, 0.1 μM dexamethasone, 2% (v/v) B27 supplement, and 25 μg/ml gentamicin. Maintenance medium was exchanged daily for the duration of the experiment.

*iCell Neurons* (Sirenko et al. 2014): cryopreserved cells were thawed and plated according to the protocol provided by the Cellular Dynamics International. Briefly, cells were plated on poly-d-lysine precoated 384-well plates (Greiner-Bio, Ref#: 781946) with iCell Neural Base Medium (Catelog#: M1010) added with iCell Neural Supplement A (Catalog#: M1032) and 3.3 mg/mL of laminin. Cells were plated at densities of 7,500 cells/well. Plates were initially kept at room temperature for 30 min before transferring to an incubator set at 37°C and 5% CO_2_ for 48 hours until assay day.

*iCell Cardiomyocytes* (Grimm et al. 2016): 384-well microplates were precoated with 25 μL 0.1% (w/v) gelatin solution per well for 2 h at 37°C and 5% CO_2_. Cryopreserved cells were thawed according to manufacturer’s instruction using iCell cardiomyocytes plating medium with 1:500 (v/v) penicillin/streptomycin. Cell suspension was diluted in plate medium to provide a final cell concentration of 2 × 10^5^ cells/mL. Subsequently, the gelatin solution was aspirated from the plates and 25 μL cell suspension was added to each well, making the final cell plating density at 5000 viable cells/well. Plates were kept at room temperature for 30 min before they were incubated at 37°C and 5% CO_2_. 48 h following cell seeding, the plating medium was exchanged with 40 μL of maintenance medium containing 1:500 penicillin/streptomycin. Maintenance medium was subsequently changed every other day for another 12 days until assay day.

*iCell Endothelial cells* (Iwata et al. 2017): Endothelial cells were plated and expanded on T-75 tissue culture flasks coated with human fibronectin solution at 3 μg/cm^2^. Cells were cultured with maintenance medium containing the VascuLife VEGF Medium Complete Kit (SKU: LL-0003), with FBS, and iCell Endothelial cells medium supplement. Cell density was determined using Trypan Blue exclusion test and a cell suspension was prepared that results in 1.0 × 10^4^ cells/cm^2^. The fibronectin solution was aspirated and cells were seeded in a T-75 flask. Cells were incubated at 37°C and 5% CO_2_ with media changes every 2 days and passaged every 3–4 days by TrypLE Express. Experiments were conducted with cells between passages 1 and 5. Cells were transferred into 384-well plates with 50 μL maintenance medium with density at 750 cells/well for cytotoxicity assay and 7,500 cells for angiogenesis assay. Cells were kept in microplates for 2-3 days until monolayer formed before adding chemicals for cytotoxicity assays.

*Human Umbilical Vein Endothelial Cells* (Iwata et al. 2017): HUVECs were plated and expanded on T-75 tissue culture flasks coated with 0.1% (w/v) gelatin solution. The culture medium contains Medium 199 with the EGM-2 BulletKit (Lonza, Catalog#: CC-3162). HUVECs were incubated at 37°C and 5% CO_2_ and passaged every 2–3 days using TrypLE Express. Cell density was determined by cell counting with Trypan Blue. Experiments were performed with cells between passages 1 and 5. Cells were transferred into 384-well plates with 50 μL maintenance medium with density at 750 cells/well for cytotoxicity assay and 3,500 cells for angiogenesis assay. Cells were kept in microplates for 2-3 days until monolayer formed before adding chemicals for cytotoxicity assays.

References

- Grimm FA, Iwata Y, Sirenko O, et al. (2016) A chemical–biological similarity-based grouping of complex substances as a prototype approach for evaluating chemical alternatives. Green Chemistry 18(16):4407-4419.
- Iwata Y, Klaren WD, Lebakken CS, Grimm FA, Rusyn I (2017) High-content assay multiplexing for vascular toxicity screening in induced pluripotent stem cell-derived endothelial cells and human umbilical vein endothelial cells. Assay and drug development technologies 15(6):267-279.
- Sirenko O, Hesley J, Rusyn I, Cromwell EF (2014) High-content high-throughput assays for characterizing the viability and morphology of human iPSC-derived neuronal cultures. Assay and drug development technologies 12(9-10):536-547.

**Text S2: ATP production of iCell Neurons and HUVECs.**

Production of ATP in iCell neurons and HUVECs were measured using CellTiter-Glo® Luminescent Cell Viability Assay according to manufacturer’s introduction. In detail, after high content imaging process, equal volume of pre-equilibrate CellTiter-Glo reagent were added into each well in assay plates. Then mixing contents for 2 min on an orbital shaker to induce cell lysis and allow the plates to incubate at room temperature for 10 min to stabilize luminescent signal. Luminescence was read using FLIPR tetra (Molecular Devices) instrument, with a read time interval of 1 second per well. Quantitative data was exported for concentration-response profiling.

**Text S3: Calcium flux assay of iCell Cardiomyocytes**.

Intracellular calcium flux in iCell cardiomyocytes exposed to the test solutions for 15 and 90 min was measured using FLIPR tetra (Molecular Devices) instrument using EarlyTox^TM^ Cardiotoxicity Kit as described in previous study (Grimm et al. 2016). Cardiomyocytes were incubated for 2 hours at 37 °C after the addition of one volume of pre-equilibrated calcium-dye reagent. Prior to exposure to test solutions, baseline calcium flux measurements were recorded at 515-575 nm following excitation at 470-495 nm and at a frequency of 8 Hz for 100 seconds. The internal instrument temperature was regulated at 37°C. Cells were then simultaneously exposed to test solutions using the internal fluidics handling system. 15- and 90-min post-exposure, the beating of cardiomyocytes was monitored as described above. Between measurements, cells were incubated under cell culture conditions at 37°C and 5% CO_2_. Recorded data were further analyzed in Screenworks 4.0 software (Molecular Devices LLC., Sunnyvale, CA) for peak processing and statistical parameters were exported as Microsoft Excel files for concentration-response assessment.

**Text S4: Angiogenesis of iCell Endothelial cells and HUVECs.**

Angiogenic assays were performed using Geltrex LDEV-Free Reduced Growth Factor Basement Membrane for both iCell endothelial cells and HUVECs in 384-well format according to previous study (Iwata et al. 2017). iCell endothelial cells were incubated with VascuLife® Basal Medium containing 4 nM L-glutamine LifeFactor and 0.1% iCell Endothelial Cells Medium Supplement. HUVECs were incubated with Medium 199 containing the EGM-2 BulletKits at 2X concentration, also the VEGF component was replaced with 12.5 ng/mL VEGF, and this was referred to as “2 X Assay Medium.” Geltrex was thawed at 4°C and dispensed to coat the plates (10 μL/well) on the ice. The plates were incubated for 1 h at 37°C. Following the incubation, a 2X chemical working solution (25 μL/well), prepared in basal medium, was added to the plate and cells resuspended in 2X assay medium (25 μL/well) were seeded at the density of 7,500 (iCell-ECs) or 3,500 (HUVECs) cells/well. Cells were exposed to chemicals overnight at 37°C at 5% CO2 and stained with Calcein AM (25 μL/well, 6 μmol/L) for 15min and processed to live cell high-content imaging.

**Table S1.** Summary of the quality control parameters evaluated for each cell type and phenotype.

| **Cell Type** | **Phenotype** | **CV%**  **Medium** | **CV%**  **DMSO** | **t-test**  **p-value** | **Intra-plate replicates (n=60)** | | | | **Inter-plate replicates (n=210)** | | | |
| --- | --- | --- | --- | --- | --- | --- | --- | --- | --- | --- | --- | --- |
|  |  |  |  |  | **Pearson (r)** | **p-value** | **Spearman (ρ)** | **p-value** | **Pearson (r)** | **p-value** | **Spearman (ρ)** | **p-value** |
| **iCell Hepatocytes** | Cell Number | 5.15 | 4.34 | 0.70 | 0.84 | <0.0001 | 0.52 | <0.0001 | 0.84 | <0.0001 | 0.36 | <0.0001 |
|  | Nuclei Intensity | 2.77 | 2.07 | 0.05 | 0.84 | <0.0001 | 0.30 | 0.02 | 0.69 | <0.0001 | 0.37 | <0.0001 |
|  | All Cell Mean Area | 8.86 | 11.58 | 0.22 | 0.27 | 0.03 | 0.01 | 0.94 | 0.42 | <0.0001 | 0.34 | <0.0001 |
|  | Mitochondrial Intensity | 10.32 | 13.31 | 0.33 | 0.40 | 0.00 | -0.01 | 0.94 | 0.46 | <0.0001 | 0.31 | <0.0001 |
|  | Mitochondrial Integrity | 4.64 | 4.18 | 0.32 | 0.79 | <0.0001 | 0.10 | 0.43 | 0.83 | <0.0001 | 0.36 | <0.0001 |
| **iCell Neurons** | Cell Number | 8.68 | 12.42 | 0.34 | -0.15 | 0.25 | -0.33 | 0.01 | 0.77 | <0.0001 | 0.44 | <0.0001 |
|  | Total Outgrowth | 12.50 | 17.93 | 0.80 | 0.35 | 0.01 | 0.20 | 0.13 | 0.75 | <0.0001 | 0.52 | <0.0001 |
|  | Mean Outgrowth | 12.26 | 12.33 | 0.10 | 0.32 | 0.01 | 0.19 | 0.14 | 0.73 | <0.0001 | 0.43 | <0.0001 |
|  | Total Process | 11.15 | 12.82 | 0.78 | -0.07 | 0.60 | -0.30 | 0.02 | 0.75 | <0.0001 | 0.38 | <0.0001 |
|  | Total Branches | 27.44 | 26.82 | 0.93 | 0.18 | 0.17 | 0.17 | 0.20 | 0.60 | <0.0001 | 0.47 | <0.0001 |
|  | Total Cell Body Area | 8.63 | 9.46 | 0.02 | -0.08 | 0.55 | -0.21 | 0.11 | 0.78 | <0.0001 | 0.37 | <0.0001 |
|  | Cell with Significant Growth | 8.67 | 12.64 | 0.33 | -0.15 | 0.24 | -0.34 | 0.01 | 0.77 | <0.0001 | 0.44 | <0.0001 |
|  | Cytoplasmic Integrity | 11.15 | 14.59 | 0.61 | -0.08 | 0.55 | -0.32 | 0.01 | 0.75 | <0.0001 | 0.42 | <0.0001 |
|  | Mitochondrial Integrity | 10.78 | 15.25 | 0.22 | -0.03 | 0.84 | -0.31 | 0.02 | 0.71 | <0.0001 | 0.41 | <0.0001 |
|  | ATP | 15.09 | 10.75 | 0.88 | 0.50 | <0.0001 | 0.14 | 0.28 | 0.85 | <0.0001 | 0.68 | <0.0001 |
| **iCell Cardio-myocytes** | Beats per minute_15min | 18.11 | 14.44 | <0.01 | 0.75 | <0.0001 | 0.54 | <0.0001 | 0.88 | <0.0001 | 0.67 | <0.0001 |
|  | Beats per minute_90min | 14.68 | 14.40 | 0.48 | 0.83 | <0.0001 | 0.65 | <0.0001 | 0.86 | <0.0001 | 0.70 | <0.0001 |
|  | Cell Number | 9.45 | 8.05 | 0.05 | 0.59 | <0.0001 | 0.15 | 0.25 | 0.71 | <0.0001 | 0.54 | <0.0001 |
|  | Mitochondrial Integrity | 11.36 | 10.46 | 0.03 | 0.57 | <0.0001 | 0.16 | 0.22 | 0.71 | <0.0001 | 0.58 | <0.0001 |
|  | Peak Amplitute_15min | 17.56 | 17.05 | 0.25 | 0.82 | <0.0001 | 0.31 | 0.01 | 0.89 | <0.0001 | 0.67 | <0.0001 |
|  | Peak Amplitute_90min | 16.38 | 15.12 | 0.29 | 0.80 | <0.0001 | 0.33 | 0.01 | 0.87 | <0.0001 | 0.65 | <0.0001 |
|  | Peak Spacing_15min | 13.03 | 16.37 | <0.01 | 0.76 | <0.0001 | 0.40 | 0.00 | 0.86 | <0.0001 | 0.64 | <0.0001 |
|  | Peak Spacing_90min | 13.28 | 11.98 | 0.68 | 0.86 | <0.0001 | 0.55 | <0.0001 | 0.60 | <0.0001 | 0.67 | <0.0001 |
|  | Peak Width_15min | 15.56 | 18.51 | <0.01 | 0.70 | <0.0001 | 0.40 | 0.00 | 0.82 | <0.0001 | 0.65 | <0.0001 |
|  | Peak Width_90min | 16.58 | 15.80 | 0.47 | 0.72 | <0.0001 | 0.53 | <0.0001 | 0.82 | <0.0001 | 0.68 | <0.0001 |
|  | Peak Rise time_15min | 8.84 | 9.16 | 0.01 | 0.90 | <0.0001 | 0.23 | 0.07 | 0.88 | <0.0001 | 0.51 | <0.0001 |
|  | Peak Rise time_90min | 8.57 | 8.07 | 0.18 | 0.85 | <0.0001 | 0.42 | 0.00 | 0.88 | <0.0001 | 0.54 | <0.0001 |
|  | Peak Decay time_15min | 16.77 | 19.81 | <0.01 | 0.68 | <0.0001 | 0.40 | 0.00 | 0.81 | <0.0001 | 0.66 | <0.0001 |
|  | Peak Decay time_90min | 18.66 | 17.70 | 0.42 | 0.68 | <0.0001 | 0.53 | <0.0001 | 0.80 | <0.0001 | 0.69 | <0.0001 |
|  | Decay to Rise Ratio_15min | 18.61 | 16.73 | <0.01 | 0.67 | <0.0001 | 0.32 | 0.01 | 0.83 | <0.0001 | 0.69 | <0.0001 |
|  | Decay to Rise Ratio_90min | 19.38 | 16.74 | 0.14 | 0.65 | <0.0001 | 0.45 | 0.00 | 0.80 | <0.0001 | 0.71 | <0.0001 |
| **iCell Endothelial Cells** | Cell Number | 8.24 | 8.67 | 0.20 | 0.58 | <0.0001 | 0.29 | 0.02 | 0.79 | <0.0001 | 0.34 | <0.0001 |
|  | Mitochondrial Integrity | 8.02 | 8.72 | 0.34 | 0.77 | <0.0001 | 0.28 | 0.03 | 0.86 | <0.0001 | 0.33 | <0.0001 |
|  | Nuclei Mean Area | 2.72 | 3.03 | 0.16 | 0.07 | 0.59 | 0.13 | 0.31 | 0.62 | <0.0001 | 0.18 | 0.01 |
|  | Mitochondrial Intensity | 13.12 | 8.03 | <0.01 | 0.70 | <0.0001 | 0.37 | 0.00 | 0.62 | <0.0001 | 0.36 | <0.0001 |
|  | Cytoplasmic Integrity | 11.81 | 9.76 | 0.06 | 0.53 | <0.0001 | 0.33 | 0.01 | 0.71 | <0.0001 | 0.30 | <0.0001 |
|  | Total Tube Length | 19.55 | 13.51 | <0.01 | 0.63 | <0.0001 | 0.37 | 0.00 | 0.60 | <0.0001 | 0.57 | <0.0001 |
|  | Mean Tube Length | 6.81 | 5.36 | 0.42 | 0.63 | <0.0001 | 0.39 | 0.00 | 0.26 | 0.00 | 0.32 | <0.0001 |
|  | Total Tube Area | 20.85 | 14.06 | <0.01 | 0.62 | <0.0001 | 0.41 | 0.00 | 0.61 | <0.0001 | 0.61 | <0.0001 |
| **HUVECs** | Cell Number | 7.05 | 6.27 | 0.19 | 0.61 | <0.0001 | 0.37 | 0.00 | 0.79 | <0.0001 | 0.33 | <0.0001 |
|  | Mitochondrial Integrity | 6.95 | 6.06 | 0.11 | 0.66 | <0.0001 | 0.37 | 0.00 | 0.82 | <0.0001 | 0.36 | <0.0001 |
|  | Nuclei Mean Area | 3.15 | 2.80 | <0.01 | 0.95 | <0.0001 | 0.49 | <0.0001 | 0.98 | <0.0001 | 0.36 | <0.0001 |
|  | Mitochondrial Intensity | 12.57 | 6.38 | <0.01 | 0.83 | <0.0001 | 0.29 | 0.02 | 0.78 | <0.0001 | 0.31 | <0.0001 |
|  | Cytoplasmic Integrity | 7.04 | 6.24 | 0.18 | 0.63 | <0.0001 | 0.37 | 0.00 | 0.85 | <0.0001 | 0.38 | <0.0001 |
|  | Total Tube Length | 13.36 | 8.77 | 0.28 | 0.63 | <0.0001 | 0.27 | 0.04 | 0.61 | <0.0001 | 0.61 | <0.0001 |
|  | Mean Tube Length | 6.62 | 5.90 | <0.01 | 0.77 | <0.0001 | 0.10 | 0.43 | 0.76 | <0.0001 | 0.39 | <0.0001 |
|  | Total Tube Area | 10.90 | 8.51 | 0.63 | 0.74 | <0.0001 | 0.29 | 0.02 | 0.62 | <0.0001 | 0.56 | <0.0001 |
|  | ATP | 2.88 | 5.20 | 0.82 | 0.88 | <0.0001 | 0.07 | 0.61 | 1.00 | <0.0001 | 0.99 | <0.0001 |

**Table S2.** EC_50_ values (μM) of positive controls in five tested cell types.

| **Cell Type** | **Phenotype** | **TAB^(a)^** | **Doxorubicin (10) ^b^** | **Brefeldin A (10)** | **Mitomycin C (100)** | **Retinoic acid (250)** | **Rotenone (50)** | **Cisapride (10)** | **Propranolol (50)** | **Isoproterenol (10)** | **Nocodazole (20)** | **Suramin (100)** | **Chloroquine(1000)** | **Histamine (400)** |
| --- | --- | --- | --- | --- | --- | --- | --- | --- | --- | --- | --- | --- | --- | --- |
| **iCell Hepatocytes** | Cell Number | 1.58 | 0.29 |  |  |  |  |  |  |  |  |  |  |  |
|  | Nuclei Intensity | 77.53 | 2.34 |  |  |  |  |  |  |  |  |  |  |  |
|  | All Cell Mean Area | 85.13 | 0.62 |  |  |  |  |  |  |  |  |  |  |  |
|  | Mitochondrial Intensity | 58.40 | 0.32 |  |  |  |  |  |  |  |  |  |  |  |
|  | Mitochondrial Integrity | 1.67 | 0.31 |  |  |  |  |  |  |  |  |  |  |  |
| **iCell Neurons** | Cell Number | 0.00 |  | NA^(c)^ | 4.36 | NA | 8.50 |  |  |  |  |  |  |  |
|  | Total Outgrowth | 0.00 |  | 0.22 | 1.93 | NA | 2.60 |  |  |  |  |  |  |  |
|  | Mean Outgrowth | 0.00 |  | 0.22 | 6.50 | NA | 7.39 |  |  |  |  |  |  |  |
|  | Total Process | 0.00 |  | NA | 3.57 | NA | 6.48 |  |  |  |  |  |  |  |
|  | Total Branches | 0.00 |  | 0.06 | 1.66 | 221.30 | 2.40 |  |  |  |  |  |  |  |
|  | Total Cell Body Area | 0.00 |  | NA | 5.31 | NA | 8.84 |  |  |  |  |  |  |  |
|  | Cell with Significant Growth | 0.00 |  | NA | 3.64 | NA | 7.86 |  |  |  |  |  |  |  |
|  | Cytoplasmic Integrity | 0.00 |  | NA | 3.61 | NA | 6.02 |  |  |  |  |  |  |  |
|  | Mitochondrial Integrity | 1.79 |  | NA | 6.23 | NA | 6.01 |  |  |  |  |  |  |  |
|  | ATP | 1.57 |  | NA | 0.73 | 241.80 | 0.82 |  |  |  |  |  |  |  |
| **iCell Cardiomyocytes** | Beats per minute_15min | 0.00 |  |  |  |  |  | 0.01 | 1.29 | 0.08 |  |  |  |  |
|  | Beats per minute_90min | 0.00 |  |  |  |  |  | 0.73 | 1.95 | 0.13 |  |  |  |  |
|  | Cell Number | 5.83 |  |  |  |  |  | NA | NA | NA |  |  |  |  |
|  | Mitochondrial Integrity | 4.48 |  |  |  |  |  | NA | NA | NA |  |  |  |  |
|  | Peak amplitute_15min | 0.00 |  |  |  |  |  | 0.00 | 1.81 | 0.02 |  |  |  |  |
|  | Peak Amplitute_90min | 0.00 |  |  |  |  |  | 0.05 | 6.56 | 0.06 |  |  |  |  |
|  | Peak Spacing_15min | 0.00 |  |  |  |  |  | 0.00 | 1.27 | 0.03 |  |  |  |  |
|  | Peak Spacing_90min | 0.00 |  |  |  |  |  | 8.73 | 2.14 | 0.55 |  |  |  |  |
|  | Peak Width_15min | 0.00 |  |  |  |  |  | 0.00 | 0.33 | 0.03 |  |  |  |  |
|  | Peak Width_90min | 0.00 |  |  |  |  |  | NA | 43.29 | 0.69 |  |  |  |  |
|  | Peak Rise time_15min | 0.00 |  |  |  |  |  | 0.01 | 3.02 | NA |  |  |  |  |
|  | Peak Rise time_90min | 0.00 |  |  |  |  |  | 1.00 | 25.25 | NA |  |  |  |  |
|  | Peak Decay time_15min | 0.00 |  |  |  |  |  | NA | 0.17 | 0.03 |  |  |  |  |
|  | Peak Decay time_90min | 0.00 |  |  |  |  |  | NA | 44.63 | 0.47 |  |  |  |  |
|  | Decay to Rise Ratio_15min | 0.00 |  |  |  |  |  | 0.01 | 11.56 | 0.07 |  |  |  |  |
|  | Decay to Rise Ratio_90min | 0.00 |  |  |  |  |  | 1.26 | 6.43 | 0.12 |  |  |  |  |
| **iCell Endothelial Cells** | Cell Number | 54.44 |  |  |  |  |  |  |  |  | 0.43 | NA | 68.96 | NA |
|  | Mitochondrial Integrity | 0.41 |  |  |  |  |  |  |  |  | 0.26 | NA | 59.39 | NA |
|  | Nuclei Mean Area | 66.77 |  |  |  |  |  |  |  |  | NA | NA | NA | NA |
|  | Mitochondrial Intensity | 68.61 |  |  |  |  |  |  |  |  | 0.12 | NA | 83.49 | NA |
|  | Cytoplasmic Integrity | 39.17 |  |  |  |  |  |  |  |  | NA | NA | NA | NA |
|  | Total Tube Length | 0.00 |  |  |  |  |  |  |  |  | 0.00 | NA | 5.93 | 0.20 |
|  | Mean Tube Length | 0.00 |  |  |  |  |  |  |  |  | 2.03 | NA | NA | 86.04 |
|  | Total Tube Area | 0.00 |  |  |  |  |  |  |  |  | 0.00 | NA | 43.33 | 0.25 |
| **HUVECs** | Cell Number | 84.79 |  |  |  |  |  |  |  |  | 5.26 | NA | 76.52 | NA |
|  | Mitochondrial Integrity | 37.14 |  |  |  |  |  |  |  |  | 4.70 | NA | 70.35 | NA |
|  | Nuclei Mean Area | 44.70 |  |  |  |  |  |  |  |  | NA | NA | NA | NA |
|  | Mitochondrial Intensity | 44.38 |  |  |  |  |  |  |  |  | 5.01 | NA | 61.31 | NA |
|  | Cytoplasmic Integrity | 0.02 |  |  |  |  |  |  |  |  | NA | NA | NA | NA |
|  | Total Tube Length | 0.00 |  |  |  |  |  |  |  |  | 0.10 | 4.73 | 395.90 | 365.00 |
|  | Mean Tube Length | 0.00 |  |  |  |  |  |  |  |  | 0.29 | 23.20 | 665.30 | 67.85 |
|  | Total Tube Area | 0.00 |  |  |  |  |  |  |  |  | 0.08 | 5.28 | 172.60 | 187.80 |
|  | ATP | 8.27 |  |  |  |  |  |  |  |  | NA | NA | NA | NA |

(a) TAB=Tetra-octyl ammonium bromide (50 μM), cytotoxicity control, values are response (%) normalized to vehicle control.

(b) Highest concentrations tested in the experiments (μM).

(c) EC_50_ value could not be derived.

**Table S3.** Overlap in the chemicals tested in different *in vivo* and *in vitro* datasets. “1” indicates the chemical was present in the dataset. “0” indicated it was not included in the dataset.

| **Chemical** | **This study** | **ToxCast** | **POD_RfD_** | **Paul Friedman et al. (2020)** |
| --- | --- | --- | --- | --- |
| Dibutyl phthalate | 1 | 1 | 1 | 1 |
| Di(2-ethylhexyl) phthalate | 1 | 1 | 1 | 0 |
| 2-Methyl-4,6-dinitrophenol | 1 | 1 | 0 | 0 |
| 1,2,3-Trichlorobenzene | 1 | 1 | 0 | 0 |
| Pentachlorophenol | 1 | 1 | 0 | 0 |
| p-Cresol | 1 | 1 | 1 | 1 |
| Benzidine | 1 | 1 | 0 | 0 |
| 2,4,5-Trichlorophenol | 1 | 1 | 1 | 1 |
| 2,4,6-Trichlorophenol | 1 | 1 | 0 | 0 |
| 2,4-Dinitrotoluene | 1 | 1 | 1 | 1 |
| Methoxychlor | 1 | 1 | 1 | 0 |
| Endosulfan | 1 | 1 | 1 | 0 |
| Dieldrin | 1 | 1 | 1 | 1 |
| Dicofol | 1 | 1 | 1 | 0 |
| Heptachlor | 1 | 1 | 1 | 1 |
| Aldrin | 1 | 1 | 1 | 1 |
| p,p'-DDD | 1 | 1 | 1 | 1 |
| Chlorpyrifos | 1 | 1 | 1 | 1 |
| o,p'-DDT | 1 | 1 | 0 | 0 |
| Azinphos-methyl | 1 | 1 | 1 | 1 |
| Dichlorodiphenyltrichloroethane | 1 | 1 | 1 | 1 |
| Trifluralin | 1 | 1 | 0 | 0 |
| 2,4-Dinitrophenol | 1 | 1 | 1 | 1 |
| Diazinon | 1 | 1 | 0 | 0 |
| Lindane | 1 | 1 | 1 | 0 |
| Parathion | 1 | 1 | 1 | 0 |
| Endrin | 1 | 1 | 1 | 1 |
| Ethion | 1 | 1 | 1 | 1 |
| Disulfoton | 1 | 1 | 0 | 0 |
| Heptachlor epoxide | 1 | 1 | 1 | 0 |
| Fluoranthene | 1 | 1 | 1 | 1 |
| Benzo(b)fluoranthene | 1 | 1 | 1 | 0 |
| Acenaphthene | 1 | 1 | 1 | 1 |
| Naphthalene | 1 | 1 | 0 | 0 |
| Benzo(a)anthracene | 1 | 1 | 0 | 0 |
| Cadmium chloride | 1 | 1 | 0 | 0 |
| Nickel(II) chloride | 1 | 1 | 0 | 0 |
| Cobalt chloride | 1 | 1 | 0 | 0 |
| Mercuric chloride | 1 | 1 | 0 | 0 |
| Zinc chloride | 1 | 1 | 0 | 0 |
| Lead nitrate | 1 | 1 | 0 | 0 |
| Potassium chromate(VI) | 1 | 1 | 0 | 0 |

**Table S4:** Detailed descriptions of each phenotype evaluated in each tested cell type.

| **Cell Type** | **Phenotype** | **Description** |
| --- | --- | --- |
| **iCell Hepatocytes** | Cell Number | Number of cell bodies in the image |
|  | Nuclei Intensity | Average area of nucleus for all cells found in the image |
|  | All Cell Mean Area | Average area of the cell (nucleus + cytoplasm) for all cells found in the image |
|  | Mitochondrial Intensity | Total pixel intensity of MitoTracker stain over the stained area in positive cells, divided by the number of cells positive for MitoTracker stain |
|  | Mitochondrial Integrity | Total number of cells positive for MitoTracker staining |
| **iCell Neurons** | Cell Number | Number of cell bodies in the image |
|  | Total Outgrowth | Total length of skeletonized outgrowth |
|  | Mean Outgrowth | Average skeletonized outgrowth divided by the number of cells |
|  | Total Process | Number of outgrowths in the image that are connected to cell bodies |
|  | Total Branches | Total number of branching junctions in the image |
|  | Total Cell Body Area | Total area of the cell bodies in the image (excluding outgrowths) |
|  | Cell with Significant Growth | Number of cells in the image with outgrowth greater than the threshold length specified in the settings |
|  | Cytoplasmic Integrity | Total number of cells positive for Calcein AM staining |
|  | Mitochondria Integrity | Total number of cells positive for MitoTracker staining |
|  | ATP | Luminescence readouts from CellTiterGlo assay |
| **iCell Cardiomyocytes** | Cell Number | Number of cell bodies in the image |
|  | Mitochondrial Integrity | Total number of cells positive for MitoTracker staining |
|  | Beats per minute | Beats per minute after exposure |
|  | Peak Amplitude | Average amplitude of peaks after exposure |
|  | Peak Spacing | Average spacing between each peak after exposure |
|  | Peak Width | Average width between each peak after exposure |
|  | Peak Rise time | Average rise time of each peak after exposure |
|  | Peak Decay time | Average decay time of each peak after exposure |
|  | Decay to Rise Ratio | Average ratio of decay to rise time of each peak after exposure |
| **iCell Endothelial Cells** and **HUVECs** | Cell Number | Number of cell bodies in the image |
|  | Mitochondrial Integrity | Total number of cells positive for MitoTracker staining |
|  | Nuclei Mean Area | The average area of nucleus for all cells found in the image |
|  | Mitochondrial Intensity | Total pixel intensity of MitoTracker stain over the stained area in positive cells, divided by the number of cells positive for MitoTracker stain |
|  | Cytoplasmic Integrity | Total number of cells positive e for Calcein AM staining |
|  | Total Tube Length | Total microns of the tube length (excluding nodes) |
|  | Mean Tube Length | Total tube length divided by the number of segments |
|  | Total Tube Area | Total square microns of tube area (excluding nodes) |
|  | ATP | Luminescence readouts from CellTiterGlo assay |

**Table S5.** ToxPi score for 42 Superfund priority list chemicals in each cell type.

| **Cell Types** | **iCell Hepatocytes** | | **iCell Neurons** | | **iCell Cardio.** | | **iCell Endo.** | | **HUVECs** | |
| --- | --- | --- | --- | --- | --- | --- | --- | --- | --- | --- |
| **Chemicals** | Min | Max | Min | Max | Min | Max | Min | Max | Min | Max |
| Benzo(a)anthracene | 0.00 | 0.00 | 0.00 | 0.60 | 0.00 | 0.98 | 0.00 | 1.00 | 0.00 | 1.00 |
| Naphthalene | 0.00 | 0.00 | 0.00 | 0.42 | 0.00 | 0.88 | 0.00 | 0.16 | 0.00 | 0.00 |
| Fluoranthene | 0.00 | 0.30 | 0.00 | 0.40 | 0.00 | 0.93 | 0.00 | 0.14 | 0.00 | 0.77 |
| Dichlorodiphenyltrichloroethane | 0.00 | 0.24 | 0.09 | 0.44 | 0.09 | 0.97 | 0.00 | 0.44 | 0.00 | 0.49 |
| Dieldrin | 0.04 | 0.25 | 0.06 | 0.30 | 0.00 | 1.00 | 0.00 | 0.74 | 0.00 | 0.53 |
| Aldrin | 0.09 | 0.22 | 0.11 | 0.46 | 0.24 | 1.00 | 0.00 | 0.49 | 0.05 | 0.73 |
| Heptachlor | 0.08 | 0.29 | 0.11 | 0.37 | 0.32 | 0.98 | 0.00 | 0.54 | 0.09 | 0.64 |
| Lindane | 0.00 | 0.78 | 0.00 | 0.47 | 0.00 | 0.59 | 0.00 | 0.73 | 0.00 | 0.00 |
| Disulfoton | 0.00 | 0.02 | 0.00 | 0.00 | 0.10 | 0.51 | 0.00 | 0.16 | 0.00 | 0.48 |
| Endrin | 0.00 | 0.08 | 0.00 | 0.00 | 0.00 | 0.97 | 0.00 | 0.52 | 0.00 | 0.59 |
| Diazinon | 0.00 | 0.19 | 0.00 | 0.38 | 0.36 | 0.78 | 0.00 | 0.63 | 0.00 | 0.69 |
| Heptachlor epoxide | 0.00 | 0.00 | 0.00 | 0.22 | 0.00 | 1.00 | 0.00 | 0.00 | 0.00 | 0.93 |
| Pentachlorophenol | 0.00 | 0.21 | 0.04 | 0.50 | 0.09 | 0.49 | 0.14 | 0.80 | 0.22 | 0.78 |
| Dibutyl phthalate | 0.00 | 0.22 | 0.00 | 0.00 | 0.11 | 0.97 | 0.00 | 0.03 | 0.00 | 0.58 |
| Chlorpyrifos | 0.00 | 0.75 | 0.08 | 0.30 | 0.00 | 0.77 | 0.00 | 0.24 | 0.00 | 0.81 |
| Di(2-ethylhexyl) phthalate | 0.00 | 0.10 | 0.28 | 0.61 | 0.00 | 1.00 | 0.00 | 0.07 | 0.00 | 0.62 |
| 2,4,6-Trichlorophenol | 0.00 | 0.06 | 0.00 | 0.32 | 0.00 | 1.00 | 0.00 | 0.40 | 0.00 | 0.36 |
| Ethion | 0.00 | 0.03 | 0.00 | 0.49 | 0.00 | 1.00 | 0.00 | 0.36 | 0.00 | 0.90 |
| Azinphos-methyl | 0.00 | 1.00 | 0.00 | 0.97 | 0.00 | 0.95 | 0.00 | 0.97 | 0.00 | 0.50 |
| 2,4,5-Trichlorophenol | 0.00 | 0.10 | 0.28 | 0.79 | 0.00 | 0.59 | 0.00 | 0.40 | 0.05 | 0.59 |
| Parathion | 0.00 | 0.49 | 0.00 | 0.67 | 0.09 | 0.62 | 0.00 | 0.16 | 0.00 | 0.88 |
| Benzo(b)fluoranthene | 0.00 | 0.25 | 0.00 | 0.10 | 0.00 | 0.69 | 0.00 | 1.00 | 0.00 | 0.55 |
| Trifluralin | 0.00 | 0.29 | 0.06 | 0.39 | 0.00 | 0.84 | 0.00 | 0.63 | 0.00 | 0.42 |
| Acenaphthene | 0.00 | 0.06 | 0.00 | 0.00 | 0.00 | 0.30 | 0.00 | 1.00 | 0.00 | 0.65 |
| p,p'-DDD | 0.10 | 0.17 | 0.08 | 0.31 | 0.09 | 0.94 | 0.09 | 0.24 | 0.05 | 0.39 |
| Benzidine | 0.00 | 0.19 | 0.00 | 0.00 | 0.00 | 0.47 | 0.00 | 0.36 | 0.00 | 0.56 |
| Endosulfan | 0.11 | 0.38 | 0.08 | 0.22 | 0.31 | 0.84 | 0.00 | 0.56 | 0.00 | 0.68 |
| Methoxychlor | 0.25 | 0.28 | 0.10 | 0.19 | 0.00 | 1.00 | 0.06 | 0.44 | 0.00 | 0.18 |
| 2,4-Dinitrophenol | 0.00 | 0.18 | 0.00 | 0.00 | 0.10 | 0.35 | 0.00 | 1.00 | 0.00 | 0.40 |
| 2,4-Dinitrotoluene | 0.00 | 0.00 | 0.00 | 0.00 | 0.00 | 0.43 | 0.00 | 0.00 | 0.00 | 0.11 |
| Dicofol | 0.11 | 0.25 | 0.07 | 0.30 | 0.09 | 0.65 | 0.03 | 0.49 | 0.07 | 0.29 |
| p-Cresol | 0.00 | 0.36 | 0.00 | 0.04 | 0.00 | 0.05 | 0.00 | 1.00 | 0.00 | 0.19 |
| o,p'-DDT | 0.00 | 0.24 | 0.09 | 0.19 | 0.08 | 0.72 | 0.00 | 0.32 | 0.00 | 0.71 |
| 2-Methyl-4,6-dinitrophenol | 0.05 | 0.89 | 0.00 | 0.33 | 0.00 | 0.42 | 0.00 | 0.23 | 0.00 | 0.58 |
| 1,2,3-Trichlorobenzene | 0.00 | 1.00 | 0.00 | 0.00 | 0.00 | 0.75 | 0.00 | 0.11 | 0.00 | 0.00 |
| Lead nitrate | 0.00 | 0.09 | 0.32 | 0.64 | 0.00 | 0.60 | 0.00 | 0.90 | 0.00 | 0.72 |
| Cadmium chloride | 0.60 | 1.00 | 0.08 | 0.29 | 0.00 | 0.89 | 0.05 | 0.25 | 0.00 | 1.00 |
| Zinc chloride | 0.00 | 0.25 | 0.00 | 0.11 | 0.00 | 0.81 | 0.00 | 0.44 | 0.00 | 1.00 |
| Mercuric chloride | 0.00 | 0.84 | 1.00 | 1.00 | 0.00 | 1.00 | 0.12 | 1.00 | 0.19 | 1.00 |
| Potassium chromate(VI) | 0.00 | 0.00 | 0.31 | 0.87 | 0.00 | 0.87 | 0.42 | 1.00 | 0.30 | 1.00 |
| Cobalt chloride | 0.00 | 0.30 | 0.00 | 0.36 | 0.00 | 0.37 | 0.00 | 0.97 | 0.00 | 1.00 |
| Nickel(II) chloride | 0.00 | 0.26 | 0.00 | 0.60 | 0.00 | 0.00 | 0.00 | 0.19 | 0.00 | 0.77 |

**Table S6.** Detailed list of the chemicals shown in the clustering diagrams (Figure 5).

| **All Cell Combined** | **ToxCast/Tox21** | **Morgan FP** | **All Cell Combined + Morgan FP** |
| --- | --- | --- | --- |
| Mercuric chloride | Lindane | 2,4−Dinitrotoluene | Azinphos−methyl |
| 2,4,5−Trichlorophenol | Azinphos−methyl | 2,4−Dinitrophenol | 2,4,5−Trichlorophenol |
| Lead nitrate | Parathion | 2−Methyl−4,6−dinitrophenol | Nickel (II) chloride |
| Potassium chromate (VI) | Diazinon | Trifluralin | Lead nitrate |
| Chlorpyrifos | Disulfoton | Lead nitrate | Chlorpyrifos |
| Azinphos−methyl | Trifluralin | Potassium chromate (VI) | Di(2−ethylhexyl) phthalate |
| Di(2−ethylhexyl) phthalate | Methoxychlor | Diazinon | Cadmium chloride |
| 2,4,6−Trichlorophenol | Dibutyl phthalate | Chlorpyrifos | Methoxychlor |
| Nickel (II) chloride | Di(2−ethylhexyl) phthalate | Parathion | Heptachlor |
| Endrin | Endosulfan | Ethion | Fluoranthene |
| Disulfoton | Dicofol | Disulfoton | Lindane |
| Acenaphthene | Potassium chromate (VI) | Dibutyl phthalate | Diazinon |
| Lindane | Cadmium chloride | Di(2−ethylhexyl) phthalate | Endrin |
| Diazinon | Lead nitrate | Azinphos−methyl | Heptachlor epoxide |
| Methoxychlor | Cobalt chloride | Fluoranthene | Aldrin |
| Dieldrin | Zinc chloride | Benzo(b)fluoranthene | Dicofol |
| Heptachlor | Nickel (II) chloride | Naphthalene | 1,2,3−Trichlorobenzene |
| Fluoranthene | Heptachlor epoxide | Acenaphthene | p,p'−DDD |
| Endosulfan | Dieldrin | Benz(a)anthracene | Naphthalene |
| Trifluralin | 2−Methyl−4,6−dinitrophenol | p−Cresol | Disulfoton |
| Ethion | Chlorpyrifos | Benzidine | Acenaphthene |
| Heptachlor epoxide | 2,4,5−Trichlorophenol | 2,4,6−Trichlorophenol | 2,4−Dinitrotoluene |
| Aldrin | Heptachlor | 2,4,5−Trichlorophenol | Parathion |
| Naphthalene | Aldrin | Pentachlorophenol | Dibutyl phthalate |
| Parathion | p,p'−DDD | 1,2,3−Trichlorobenzene | 2,4−Dinitrophenol |
| Dibutyl phthalate | o,p'−DDT | o,p'−DDT | Trifluralin |
| 2,4−Dinitrophenol | p,p'−DDT | p,p'−DDT | Cobalt chloride |
| 2,4−Dinitrotoluene | Pentachlorophenol | p,p'−DDD | o,p'−DDT |
| o,p'−DDT | Mercuric chloride | Dicofol | p,p'-DDT |
| p,p'-DDT | Fluoranthene | Methoxychlor | Ethion |
| p,p'−DDD | Endrin | Nickel (II) chloride | Benz(a)anthracene |
| Dicofol | 2,4−Dinitrotoluene | Lindane | Endosulfan |
| Benz(a)anthracene | 1,2,3−Trichlorobenzene | Endrin | Dieldrin |
| Cobalt chloride | p−Cresol | Dieldrin | Benzo(b)fluoranthene |
| Cadmium chloride | Naphthalene | Heptachlor epoxide | 2−Methyl−4,6−dinitrophenol |
| Zinc chloride | Acenaphthene | Heptachlor | p−Cresol |
| Pentachlorophenol | 2,4,6−Trichlorophenol | Aldrin | Benzidine |
| 2−Methyl−4,6−dinitrophenol | Benzidine | Endosulfan | 2,4,6−Trichlorophenol |
| 1,2,3−Trichlorobenzene | Ethion | Zinc chloride | Zinc chloride |
| Benzo(b)fluoranthene | Benzo(b)fluoranthene | Cobalt chloride | Pentachlorophenol |
| p−Cresol | Benz(a)anthracene | Mercuric chloride | Potassium chromate (VI) |
| Benzidine | 2,4−Dinitrophenol | Cadmium chloride | Mercuric chloride |


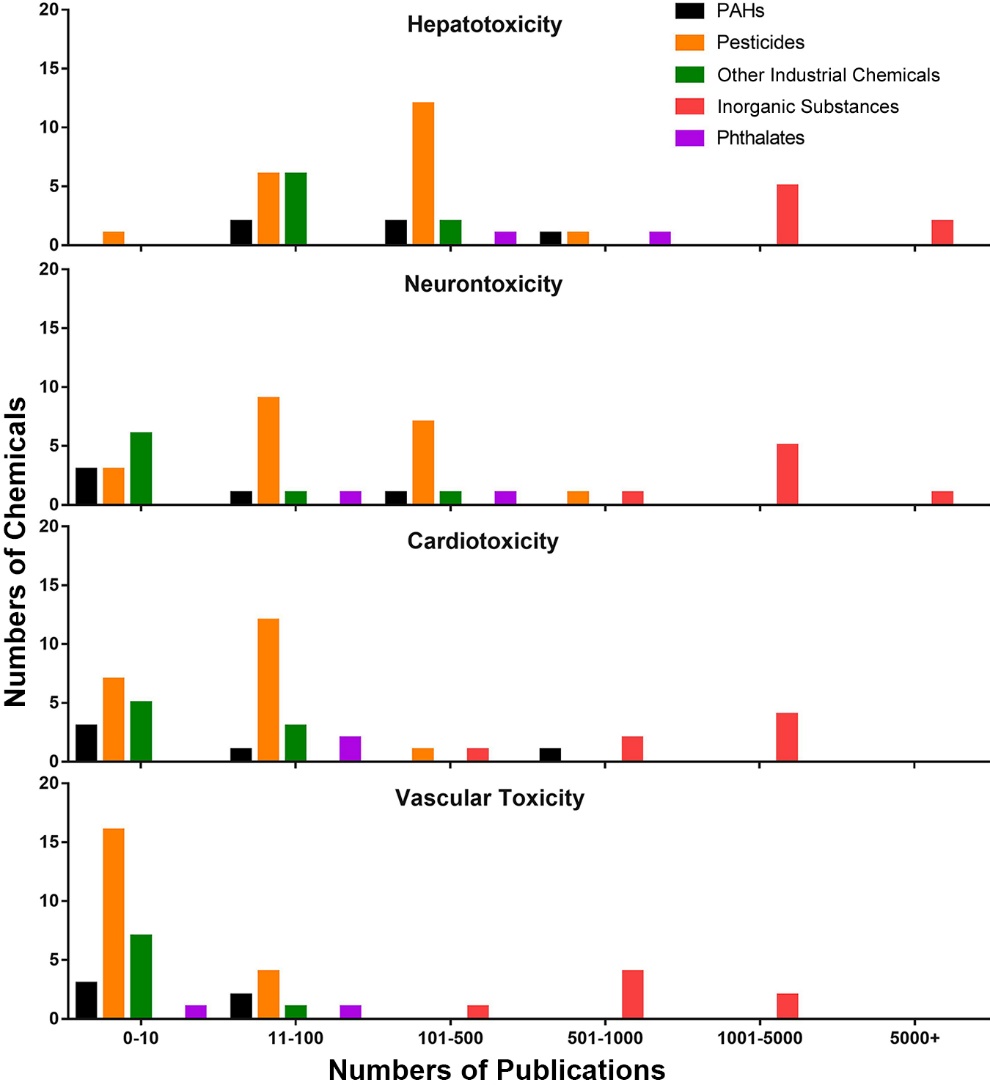


**Figure S1.** Summary of the literature review of the published evidence for the effects of the 42 Superfund priority list chemicals on various organs. The literature review is available on the Health Assessment Workspace Collaborative (Shapiro et al., 2018) web portal (<https://hawcproject.org/assessment/783>; <https://hawcproject.org/assessment/784>; <https://hawcproject.org/assessment/785>; <https://hawcproject.org/assessment/786>).


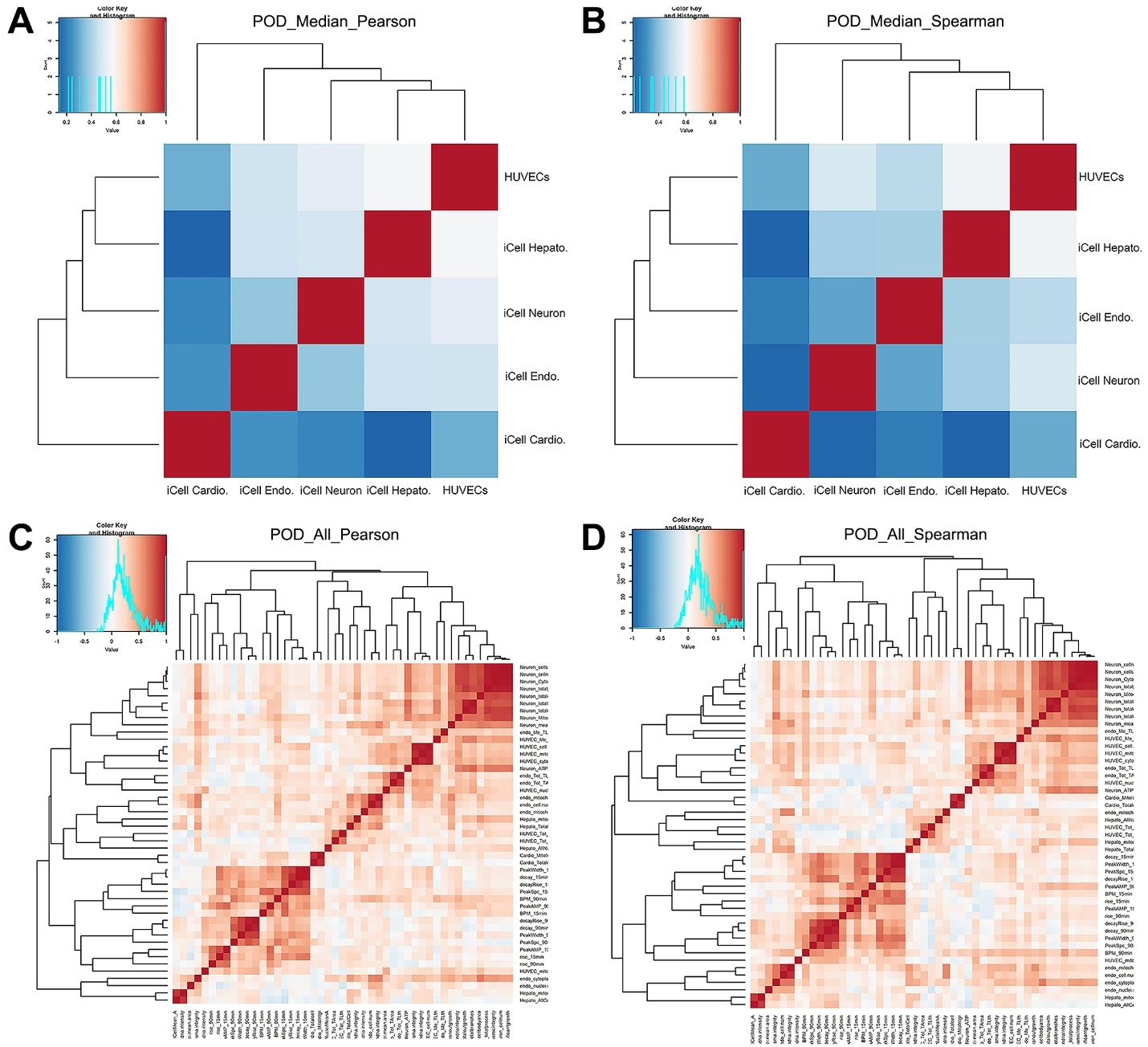


**Figure S2.** Correlation of the PODs in different cell types. Pearson (A) and Spearman (B) correlation of the POD median from each cell type are shown. Pearson (C) and Spearman (D) correlation of all PODs generated from all phenotypes of five tested cell types are shown. The color key indicates positive (red) and negative (blue) correlation values.


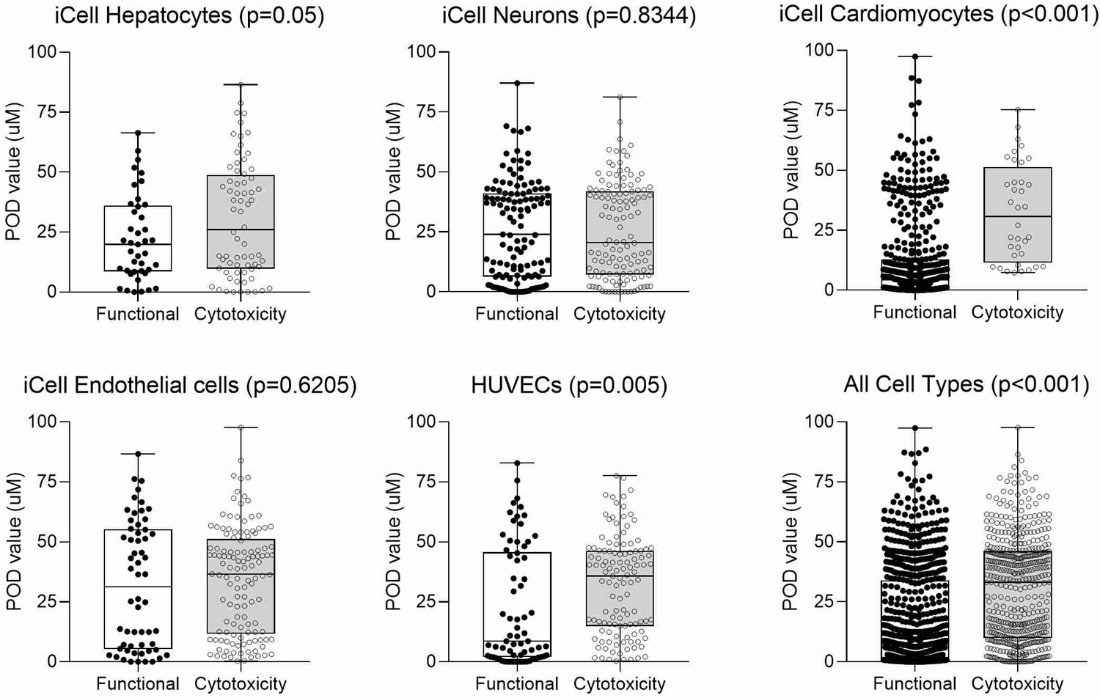


**Figure S3.** Statistical comparison of PODs generated from cytotoxicity and functional endpoints in each tested cell type. PODs for chemical/phenotype combinations that were less than the top concentration tested (100 μM) were included in the analysis. P-values shown are from unpaired t-test with Welch’s correction.


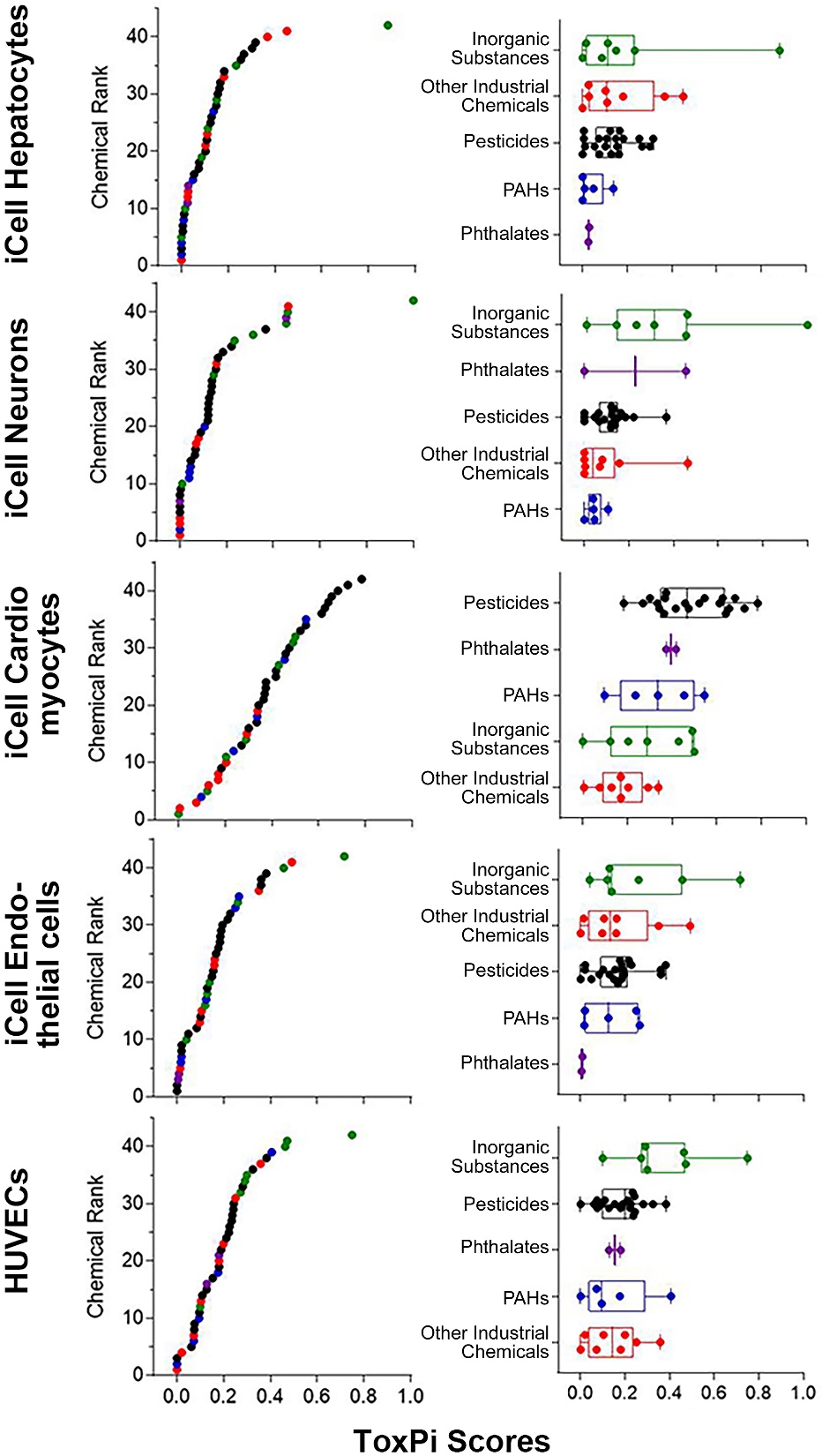


**Figure S4.** ToxPi scores of 42 Superfund priority chemicals calculated from each cell type. Each chemical (left panel) and for all chemical classes (right panel) were ranked based on each cell type. Each dot represents one chemical and the box (inter-quantile range and median) and whiskers (min to max) plots show the range of ToxPi scores.


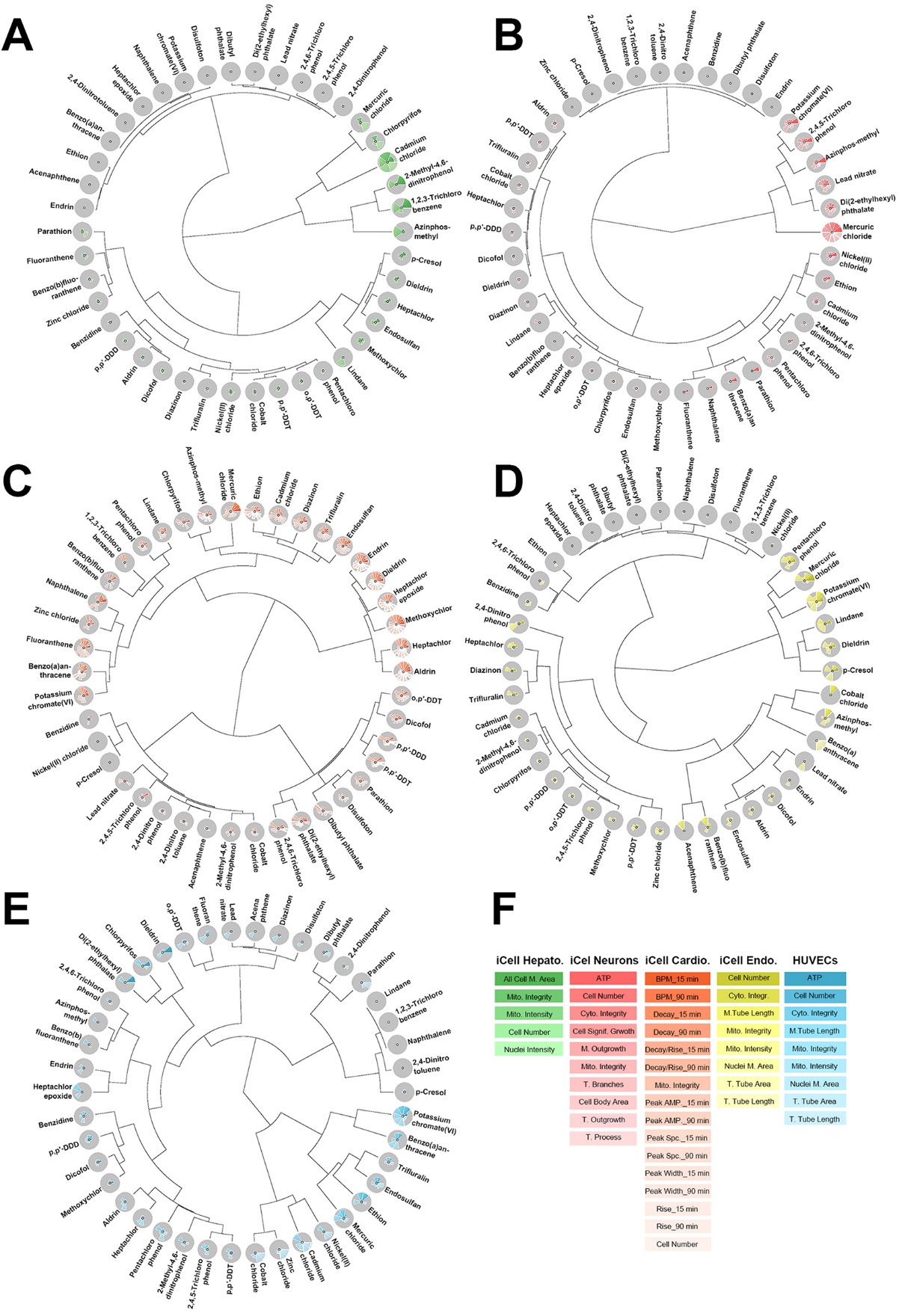


**Figure S5.** Clustering (Ward’s D method) of 42 Superfund priority list chemicals using ToxPi scores calculated from iCell hepatocytes (A), iCell neurons (B), iCell cardiomyocytes (C), iCell endothelial cells (D), and HUVECs (E). Color of each slice in ToxPi represents different phenotypes in each cell type (F).


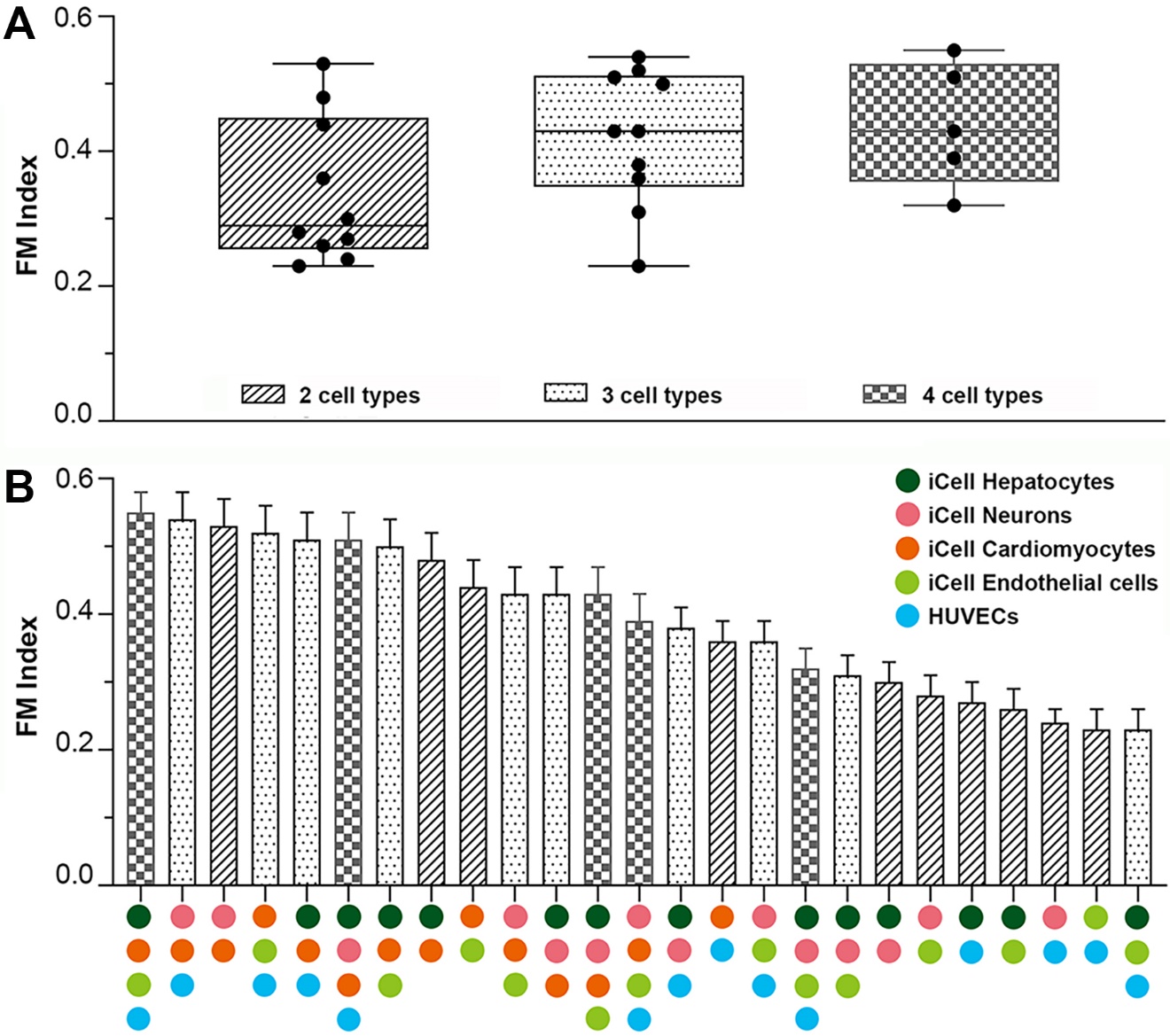


**Figure S6.** The Fowlkes-Mallows (FM) index for clustering of chemicals into 5 classes based on different combinations of cell types used in this study. (A) Box (inter-quantile range and median) and whiskers (min to max) plots indicated the overall FM indexes from the combination of 2, 3, and 4 cell types, each dot represents one specific combination, which is detailed in (B), where different combinations are ranked based on the FM index.


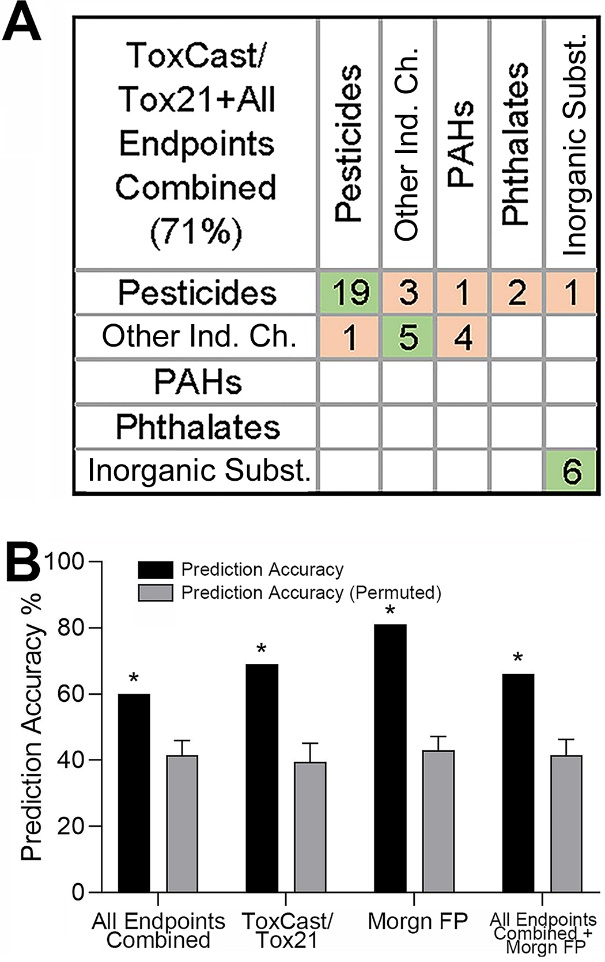


**Figure S7.** Accuracy of predicting 42 Superfund priority list chemicals into classes using the combination of *in vitro* datasets from this study and the ToxCast (A) and the comparison of prediction accuracy between biological/chemical database and permutation-based class assignment (B).
